# Supplementary material for: Data-driven design of shape-programmable magnetic soft materials
Source: Nat Commun. 2025 Mar 26;16:2946. doi: 10.1038/s41467-025-58091-z (PMC11947188; doi:10.1038/s41467-025-58091-z)
Supplement: Supplementary file 1 — Supplementary Information [file 41467_2025_58091_MOESM1_ESM.pdf]

Supplementary Information

Data-driven design of shape-programmable magnetic soft materials

Alp C. Karacakol[1,2], Yunus Alapan[1,3,4*], Sinan O. Demir[1,5], Metin Sitti[1,5,6*]

[1] Physical Intelligence Department, Max Planck Institute for Intelligent Systems, 70569 Stuttgart, Germany

[2] Department of Mechanical Engineering, Carnegie Mellon University, Pittsburgh, PA 15213, USA

[3] Department of Mechanical Engineering, University of Wisconsin-Madison, Madison, WI 53706, USA

[4] Department of Biomedical Engineering, University of Wisconsin-Madison, Madison, WI 53706, USA

[5] Stuttgart Center for Simulation Science, University of Stuttgart, 70569 Stuttgart, Germany

[6] School of Medicine and College of Engineering, Koç University, 34450 Istanbul, Turkey

[*] Correspondence to: sitti@is.mpg.de, alapan@wisc.edu

This file includes:

Supplementary notes........ 3

S1 – Data-driven design algorithm for stimuli-responsive materials........ 3

S1.1 – Representation of parameters........ 3

S1.2 – Algorithm........ 8

S1.3 – Benchmarking compared to selected MAP-elites variants........ 17

S1.4 – Algorithm runtime of the demonstrations........ 24

S2 – Simulation environment........ 25

S3 – Shape complexity score........ 27

S4 – State-of-the-art for design of stimuli responsive soft materials........ 30

S4.1 – Design of magnetically responsive soft materials........ 30

S4.2 – Design of stimuli-responsive soft materials........ 33

S5 – Performance measurements........ 35

S5.1 – Performance objective functions........ 36

S5.2 – The mathematical representations of the desired shapes for shape-matching demonstrations........ 42

S6 – Design space calculation........ 46

Supplementary figures........ 48

Supplementary tables........ 85

Supplementary references........ 92

Supplementary notes

S1 – Data-driven design algorithm for stimuli-responsive materials

We developed our data-driven design algorithm to spatially program both the structure and the stimuli-response of materials for desired behaviors with pre-defined control signal (external stimuli). Since each sub-module of our framework is established in a generic manner, the introduced approach is capable of designing multi-material and intrinsically 3D structures. Multi-material capability not only includes passive and magnetically responsive materials with various elastic modulus but also allows the utilization of different stimuli-responsive materials. This subsection describes the sub-modules of the design algorithm in detail, and summarizes the similarities and differences with the state-of-the-art in the literature.

S1.1 – Representation of parameters

The representation of the parameters of a design space is a crucial step in any design task. A good representation could enable a relatively efficient space exploration even for a high-dimensional design space by creating a bias towards potential high-performing solutions. Depending on the representation, solutions might have a tendency towards designs with simple or complex symmetries, repetitions, and variations. In the context of stimuli-responsive soft materials, parameters for the structural design and the material response programming should be both considered. Structural design includes morphology of the design and spatial distribution of material palette, whereas the material response programming refers to spatial encoding of directional material response. For some responsive materials, such as thermally expanding/contracting materials[1], these two parameters may imply the same thing as the material response is directly governed by the material type. For others, including magnetically responsive and liquid crystal elastomers (LCE), these parameters would be different as the response of the material depends on the domain alignments, such as magnetization direction or orientation of liquid crystal molecules. To be able to capture both material classes in our

framework, we decided on two parameter spaces, one representing the morphology along with the material type, and the other one for programming directional material response.

We employ Compositional Pattern-Producing Network (CPPN) [2] to represent the structural design (morphology). CPPNs are similar to neural networks in terms of the architecture of nodes with activation functions and connecting edges with weights, but, in contrast to neural networks, these components are not fixed and can be evolved over iterations. Hence, it enables fluidity in the abstraction of the morphology representation. CPPN maps the spatial coordinates of the voxels in the workspace to the type of voxel (i.e., 0 for “empty” voxels, and an integer number mapping to material type) by taking the 3D Cartesian coordinates and bias values (i.e., the distance from the center) as inputs. This indirect encoding method is shown to be capable of generating spatial symmetries, and repetitions with regularities and/or variations[2,3]. While the initial CPPN starts with a simple geometric pattern for morphology, the changes applied on the network parameters can complexify these patterns over the iterations. Representation of soft robot morphology via CPPN is shown to be a powerful method to generate coordinated material distribution over the robot morphology[4-7]. The capability to reduce parameter number for the robot morphology without compromising on the complexity of the structural design motivated us to utilize CPPN for the structural design representation in this work.

In contrast to structural design, we utilized direct encoding for programming of the material response, specifically magnetic prolife in this work, mainly due to the ineffectiveness of CPPN to generate arbitrarily changing local directional material response. The tendency of the CPPNs to generate distinct yet combined blobs of coordinated sections limit the capability to represent discretely changing material programming at local scales. The distinct and independent programming of the neighboring voxels/segments at the local scales can enable more complex robotics behaviors for stimuli-responsive soft materials as demonstrated in a wide-range of studies[8-13]. We also avoided the utilization of a monolithic CPPN, representing

both structural design and material response programming with a single CPPN, as unified CPPNs are known to experience pleiotropic effects.

Implementation of CPPN:

CPPN is created as a collection of nodes and edges without a fixed network architecture, implying that node numbers, node activation function types and edge connections can be altered. The inputs of the CPPN consists of 5 terms and are defined as the cartesian coordinates (*x, y, z*) of each voxel, a bias term (that is set to 1), and a distance term (the distance of the voxel to the segment centroid). The distance term is utilized for creating a bias to achieve repeating patterns in structural design. The input values are passed through CPPN to give a single output defining the material type for the given voxel coordinate. The inputs are normalized between 0 and 1. The output node activation function is selected as sigmoid, and the resulting value is mapped to the material type by discretizing the 0 to 1 range into the number of material types.

The CPPNs are initialized by randomly adding 5 nodes, followed by arbitrary additions of 10 edges. The weights of the edges are drawn from a uniform distribution between -1 and 1. Randomly selected 5 of the edges are removed. Then, randomly selected nodes are assigned with activation functions chosen from the set [“sin(x)”, “±abs(x)”, “±square(x)”, “±sqrt(abs(x))”, “square(sin(x))+x”, “Gaussian“, periodic functions of “triangle wave”, “square wave”, “rectified sine wave”, and traditional NN functions of “relu”, “elu”, “tanh”, “swish”] for 100 times. Finally, 100 edges are randomly selected and their weights are changed by summing with a value sampled from a normal distribution with 0 mean and 0.5 standard deviation. The resulting values for the weights are clipped between -1 and 1. After the initialization, the resulting structural designs are validated for the pre-defined conditions of minimum filled voxel ratio, and material diversity. If the resulting design does not satisfy these conditions, the initialization steps are repeated on the existing CPPN up to 1500 times. The CPPN are discarded after this point if they still do not satisfy the conditions, and a new CPPN is initialized. The hyperparameters are mostly adapted from the work of S. Kriegman et al. [5].

Other potential approaches:

In the discussion section of our manuscript, we briefly highlighted the potential advantages of exploring parameter representation methods beyond Compositional Pattern Producing Networks (CPPNs). Adaptation of more advanced methods holds promise for enhancing computational efficiency and generalization for the design of stimuli-responsive soft materials. The potential promising methods worth exploring are traditional techniques like density-based representations, inspired by the principles from topology optimization, as well as statistical methods such as Gaussian mixture models and Principal Component Analysis (PCA). Additionally, representation learning techniques[14], including autoencoders[15], Variational Autoencoders (VAEs) [16], and manifold learning methods, present intriguing possibilities. Recent literature showcases hybrid approaches that combine traditional representation methods with deep learning strategies. For instance, neural networks are utilized as implicit field functions[17] and Neural Metamaterial Networks (NMN) for encoding metamaterial structures[18] are introduced, facilitating gradient-descent optimization. The ongoing advancements in parameter representation methods are crucial for effectively reducing the dimensionality of parameters in the realm of stimuli-responsive soft material design. However, the adoption of these techniques requires further investigation and comprehensive benchmarking to ensure their effective implementation and utilization.

Choosing voxel and segment resolutions for data-driven design algorithm:

The resolution of voxels and segments influences the optimization process by affecting the dimensionality and granularity of the design space. While finer resolutions allow for more detailed and expressive designs, they also increase computational complexity and may result in solutions that are more challenging to fabricate. Conversely, coarser resolutions simplify the optimization process and improve computational efficiency but may limit the diversity or precision of the resulting designs.

In this work, these parameters are defined depending on the capabilities and/or limitations of the fabrication method along with the consideration of the computational time of the simulations as followed:

- The voxel sizes are defined as 200 μm and 2 mm for the demonstrations fabricated by heat-assisted magnetic programming and voxel-assembly methods, respectively.
    - Heat-assisted magnetic programming: A voxel size with a side length of 200 μm was used due to the thickness of our magnetic soft material, which was chosen to ensure uniform magnetization throughout the volume via heat-assisted magnetization (Fig. S21C).
    - Voxel-assembly: A voxel size with a side length of 2 mm was chosen to minimize fabrication errors and complexity, as manual gluing of smaller voxels increases artifacts and fabrication time.
- The segment size, thus voxel number within a segment, is also chosen depending on the capabilities of the fabrication methods.
    - Heat-assisted magnetic programming: The magnetization resolution of the method in our setup is at 600 μm due to the laser spot size. Considering this constraint, the segment sizes are selected at smallest 600 μm in length and width. For the magnetic soft beam demonstrations, segments with 600 μm in length and 1 mm in width, which are equivalent to the width of the beam, are employed. For the magnetic soft sheets, segments with 600 μm in length and width are used, only except for Fig. S12B, in which segments of 1 mm length and width were chosen to meet integer division requirement.
    - Voxel-assembly: Since each voxel is magnetized one by one in voxel-assembly method, the segments are equivalent to a single voxel for all the demonstrations with 2 mm voxel size.

- Total voxel numbers of the demonstrations are defined by considering either the computational evaluation time or the expected average fabrication time of the samples.
  - For 200 µm voxels, the computational evaluation time was targeted to fall within the 60–120 seconds range.
  - For 2 mm voxels, the voxel number was limited to approximately 250, based on the fabrication constraints of the voxel-assembly method. This limit assumes a fabrication time of ~1 minute per voxel for manual gluing, allowing the final design to be completed within ~6 hours.

S1.2 – Algorithm

S1.2.A – Background on MAP-elites and variants

MAP-elites is a quality diversity algorithm, which is a class of stochastic optimization methods. It utilizes an objective function, and discretizes the design space into cells representing a map of user-chosen low-dimensional features. MAP-elites searches for a design within each cell of the map that is maximizing or minimizing the given objective. The best-performing designs for the cells are saved over the iterations, resulting in an archive of designs. The MAP-elites have proven to be capable of producing high-quality and diverse solutions within highly deceptive non-convex search spaces in a single run. However, MAP-elites suffers from sample inefficiency (i.e., requires high number of evaluations), and limited high-dimensional search space scalability[19]. Novel approaches are proposed to overcome these limitations since the introduction of MAP-elites[20] by combining it with surrogate models[21-25], representation learning[26,27] or other data-efficient algorithms[28-32].

The most preferred method at improving the sample efficiency of MAP-elites is the use of surrogate models. Surrogate Assisted Illumination (SAIL) algorithm[21] utilizes Gaussian processes (GP) for modelling the objective function inspired by the Bayesian Optimization (BO). Expanding upon SAIL, Surrogate-Assisted Phenotypic Niching[22] (SPHEN) models both objective and descriptor landscapes on separate GPs, enabling black-box features. Bayesian

Optimization of Elites[23] (BOP-Elites) algorithm, is a BO algorithm tailored as a Quality Diversity (QD) method inspired by MAP-Elites and BO, surpassing the improvements shown in SPHEN and SAIL. While SAIL, SPHEN, and BOP-elites algorithms demonstrate orders of magnitude better sample efficiency compared to MAP-Elites, these methods suffer from the curse of dimensionality similar to the limitations of GP and BO. The sample inefficiency of MAP-Elites is particularly concerning for high dimensional problems with computationally expensive evaluations. In these arduous situations, computational cost of evaluations is reduced by online trained surrogate models[24,25] that can be exploited by MAP-elites. Both Dynamics-Aware Quality-Diversity[24] (DA-QD) and Deep Surrogate Assisted MAP-elites[25] (DSA-ME) highlight the benefit of online trained surrogate models on the accuracy of the predictions and sample efficiency.

Learning a representation of solutions via generative models is another promising method to scale MAP-elites to higher-dimensional problems in a data-efficient manner. Data-Driven Encoding MAP-elites[26] (DDE-elites) algorithm relies on a combined MAP-elites and Variational Autoencoder (VAE) to achieve simultaneous optimization and representation learning that is biasing MAP-elites towards high-performing solutions. Similar to DDE-elites, Policy Manifold Search[27] (PoMS) algorithm utilizes MAP-elites and Autoencoder (AE) for policy search and lower-dimensional manifold learning. Both approaches learn the solution space representation online and uses MAP-elites to search within learned latent space.

The MAP-elites commonly relies on Evolutionary Algorithm (EA) as the underlying search algorithm. Another approach to address the curse of dimensionality and high computational cost of evaluations is the utilization of other more data-efficient algorithms at the core of MAP-elites based on Evolution Strategies (ES) or gradient methods. MAP-Elites with Evolution Strategies[28] (ME-ES) scales MAP-elites to high-dimensional control tasks. Covariance Matrix Adaptation MAP-Elites[29] (CMA-ME) demonstrates significant performance enhancements in terms of quality and data-efficiency, relying on derivative-free Covariance Matrix Adaptation Evolution Strategy[33] (CMA-ES). Extending on CMA-ME, Multi-Emitter MAP-

Elites[30] (ME-MAP-Elites) improves quality, diversity and sample efficiency compared to MAP-elites and CMA-ME by leveraging the diversity of a heterogeneous set of emitters. When the evaluation functions are first order differentiable, gradient information could be leveraged for efficient exploration. MAP-Elites via a Gradient Arborescence[31] (MEGA) shows high sample efficiency by leveraging the gradients of objective and measurement functions with the introduced concept of gradient arborescence. Inspired from Deep Reinforcement Learning, Policy Gradient Assisted MAP-Elites[32] (PGA-MAP-Elites) introduces a method to efficiently achieve high performance neural network controllers by leveraging gradient estimates from an online trained critic neural network.

These advances in MAP-elites variants demonstrated the applicability of MAP-elites in wide range of fields. The majority of the works are focused on robotics, with specific applications in damage recovery[34], skill discovery[24,26,27,30,32] and neuroevolution of policy networks[28]. MAP-elites is also shown for discovery of self-assembling behaviors in swarm of microrobots[35] and scenario generation in human-robot interaction[36]. Outside of robotics, gaming industry may benefit from the automated procedural content generation[37,38], and strategic gameplay creation[25]. The diverse and high-performing exploration capability is shown to be useful for nanomaterials discovery[39] and different molecule production[40]. Lastly, efficient aerodynamic shape design[21,22] and a preliminary study on urban design[41] (ARCH-elites) highlights the potential of MAP-elites in design problems as well. While the extensive range of applications utilizing MAP-elites emphasizes a vibrant interest from diverse fields, the field of stimuli-responsive materials for designing the material programming and structure has, to our knowledge, not capitalized on this potential. The capability of MAP-elites for generating high-quality and diverse design solutions within enormous and highly deceptive design spaces inspired us to utilize MAP-elites in our work. To improve the data-efficiency of MAP-elites, we coupled it with an online trained Neural Network (NN) without prior data.

S1.2.B – Integration of MAP-elites with online trained Neural Networks for enhanced data-efficiency

MAP-elites is employed at the core of our algorithm for searching the design space, which deals with the exploitation (quality) and exploration (diversity) tradeoff by decoupling them. The exploitation is achieved by searching high-performing solutions and encouraging local competition within the cells, whereas the exploration seeks global novelty within the design archive. The exploration and exploitation modes for each iteration is selected randomly with equal probability in our implementation. The exploration mode selects random cells from map, while exploitation mode selects the best-performing cells of the map. We use Evolutionary Algorithm (EA) as the underlying optimization algorithm for MAP-elites, as it is employed in the original MAP-elites work[20]. Thus, similar to EA, the design alteration within MAP-elites is achieved by mutation and cross-over operations.

Design alterations

Mutations make exploratory stochastic variations to the given design parameters. In our work, the designs consist of spatial programming of the material response and the structural material composition. We made the mutations either on the directly represented material programming or on the structural design by changes in CPPN, the choice over which is decided randomly with equal probability. The decoupling of the mutations for these two distinct properties ensures a more gradual change in the designs, resulting in trackable improvements on either structural design or material response programming over iterations. The mutations on the structural design implemented by changes in CPPN node numbers, edge connections, and the weight of existing edges. Hence, the mutations have the freedom to tune all of the parameters of network architecture of CPPN by changing the node types or edge weights, adding/removing nodes or edge connections. When the CPPNs are mutated, 5 new nodes are added, followed up by 10 new edge additions and 5 edge removals. Then, the activation function of randomly selected 100 nodes is changed. Finally, the weights of randomly selected 100 edges are updated with Gaussian mutation (mean and standard deviation set as old

weight value, and 0.5, respectively) and clipped between -1 and 1 range. We defined the mutations on the programming of stimuli-responsive materials as randomly sampled Gaussian mutation value added on a randomly selected segment and clipped between the defined ranges ([-π, π] for $\mathbf{M}_\theta$, and [0, π] for $\mathbf{M}_\varphi$). The mutation is applied to $\mathbf{M}_\theta$ for 2D magnetic profiles and to either $\mathbf{M}_\theta$ or $\mathbf{M}_\varphi$ with equal probability for 3D magnetic profiles.

Crossovers generate new design solutions from existing best-performing designs of the cells (elites). We defined crossover operation as the interchange between the structural design (morphology) and the programming of stimuli-responsive materials (specifically, magnetic profile in this work), simply, exchanging CPPN architectures. The crossover is applied to randomly selected and coupled elites of the cells. This interchange between the elite couples ensures combinations of promising structural designs with promising material programming.

Enhancing data-efficiency

While MAP-elites is effective at generating diverse and high-performing designs in a single run, the search process within huge design spaces requires a tremendous number of evaluations in the range of millions. For example, the initial MAP-elites paper[20] required about twenty million evaluations (20e6) for generating a repertoire of robot controllers. Our simulation evaluations take ~2 minutes on average, much faster than commonly employed FEA-based evaluations in stimuli-responsive material field, for the demonstrated cases. However, a million (1e6) evaluations still take ~33000 hours or ~1389 days, which is simply impractical to operate. Thanks to the platform-agnostic C++ implementation of our simulation engine, these time spans could be reduced by taking advantage of parallelization. Parallel run of 50 simulation evaluations reduces the time span to ~667 hours or ~28 days for a million evaluations. As we want to limit the total run time of the algorithm to a feasible time-span of 7 days (1 week), a hard-limit of 2.5e5 (quarter million) is set as the total evaluation budget for all of the demonstrations shown in this work. Hence, a single run consists of 5000 total iterations with 50 simulation evaluations each iteration. This limit on the evaluation budget

(2.5e5) requires a sufficiently efficient algorithm to achieve diverse and high-performing designs.

To achieve such data-efficiency with MAP-elites, we have coupled it with a Neural Network (NN) that is trained online in parallel without prior data. We utilize the NN as a surrogate model to guide the mutations of MAP-elites, enabling better exploitation. The NN is not trained on any prior data, which make the surrogate model incompetent of educated guesses at the early iteration phase. Due to this, it is not utilized by the MAP-elites for an initial pre-defined number of 2000 iterations. After the pre-defined iterations, the MAP-elites starts generating 250 (5 times more) mutations for exploitation mode iterations compared to the initial phase. The generated designs are pre-checked within the trained NN for the promising ones, and the most-promising 50 of them is fed to the simulation for evaluation. As the iterations progresses, the surrogate model provides better estimates, which support MAP-elites to search for better performing designs within the cells. This interconnected algorithm structure with a self-reinforcing cycle gradually improves both the NN model and MAP-elites search, enabling the sample-efficient data-driven design algorithm for stimuli-responsive soft materials.

The MAP-elites relies on surrogate model only for determining on which designs should be evaluated at the simulation environment. While the online trained NN as a surrogate model gradually gets better at providing educated guesses on the design candidates, the predictions on the unobserved designs never get closer to the accuracy of the simulation environment. We observed the surrogate model cannot generalize to all areas of the enormous design space. Hence, it cannot replace the simulation environment at any point during the iterations of the algorithm. The complex design space resulting from the highly non-linear coupled physics of stimuli-responsive materials require a systematic study on the NN architectures for surrogate modeling and necessary data number for successfully replacing simulation environments.

NN architecture and training details

NN is constructed with "Keras" as fully connected neural network with a dropout ratio of 0.1. The input layer is structured as the total node number of morphology voxels and magnetic profile segments, followed by 6 dense layers with 128 nodes using "tanh" as an activation function. The final layer of 1 node for regression is added to these layers with the activation functions of "sigmoid" for shape-morphing and "Leaky ReLU" for the other demonstrations including robot behaviors. The resulting root-mean-squared-error (RMSE) of the desired positions for the shape-morphing magnetic soft beams are scaled from 0-12 mm to 0-1 range satisfying the "sigmoid" output range. Any RMSE values greater than 12 mm is clipped to 1. As the min-max range of the robotic behavioral demonstrations are not known, we use the "Leaky ReLU" at the output node without any scaling of the performance values.

The Adam optimizer is utilized for training with a default learning rate of 0.001. The loss is defined as a mean-squared error, and the batch size is set to 512. The generated results during the iterations are divided randomly into test and train data with a ratio of 10:90. The NN is trained in parallel to the simulation evaluation of the promising designs. The epoch number for each training run is adaptively adjusted to the simulation time, resulting in approximately 12 epochs on average per iteration.

Implementation of the overall algorithm:

Our algorithm starts with creating the MAP-elites design archive and the neural network architecture with the defined hyperparameters. For 2D and 3D shape-morphing demonstrations, the design archive is created in 2D (matrix) and 3D tensor format for the selected features, respectively. The selected features are the net magnetic moment direction and the filled voxel volume of the overall design. The net magnetic moment is represented with ($\mathbf{M}_{\theta}$) and ($\mathbf{M}_{\theta}$, $\mathbf{M}_{\varphi}$) for 2D and 3D magnetization directions, respectively. Hence, the features for 2D and 3D shape-morphing demonstrations result in the parameters of ($\text{Voxel}_{\text{ratio}}$, $\mathbf{M}_{\theta}$) and ($\text{Voxel}_{\text{ratio}}$, $\mathbf{M}_{\theta}$, $\mathbf{M}_{\varphi}$), respectively. The boundaries for these parameters are selected

as [0.6, 1], [-π, π], and [0, π] for $Voxel_{ratio}$, $\mathbf{M}_{\theta}$, and $\mathbf{M}_{\varphi}$, respectively. The resulting features are divided into 50, 50, and 25 equal sections for $Voxel_{ratio}$, $\mathbf{M}_{\theta}$, and $\mathbf{M}_{\varphi}$, respectively, resulting in 2500 and 62500 cells for 2D and 3D feature spaces, respectively. Then, the NN architecture is established for the defined workspace and dimensionality as explained in detail before. Finally, an initial population of 50 designs are generated.

The generated design population is evaluated within the simulation environment. The performance of the evaluated designs is recorded and the design archive, along with the NN dataset, are updated accordingly. Afterwards, a new population of designs are selected according to the mode of selection “exploit” or “explore”. The selection mode is chosen randomly with an equal probability. If the “explore” mode is selected, sampling for the new population is done by selecting the 49 cells with best novelty scores till the threshold of 500 generations (2.5e4 total evaluations) is reached. The novelty score is defined as the average distance of the cell to its nearest 15 neighboring cells and it is calculated till the threshold is reached. After this threshold, “explore” mode selects 49 random cells within the design archive. When the “exploit” mode is selected, the best performing 49 filled cells within the design archive are selected for the new population. Then, the designs of the selected population are changed through mutation and crossover operations. Among 49 design candidates, 4 of them are selected and paired randomly for crossover operations. Mutation operations are conducted on the rest of the candidates. When the total evaluation number of 1e5 is reached, the mutations are guided via online trained NN. When NN guidance is activated, each of the 45-parent design candidate is mutated 250 times in parallel to get 250 possible pseudo candidates, which are then evaluated within the NN to get performance predictions. The pseudo candidate with the best performance prediction among 250 possible pseudo candidates is chosen for the population as a new design candidate and repeated for each parent. If the population number of 49 cannot be achieved through the sampling of the design archive during explore or exploit modes, randomly initialized new design candidates are added to the population till the total number of 49 is attained. Then, 1 randomly generated

design candidate is added to the population, reaching the total number of 50 design candidates. While all of these design candidates are sent to the simulation environment for evaluating their performances, the NN is trained with the latest dataset in parallel. Then, the same procedure is repeated for a defined number of iterations.

In our framework, we have set the maximum number of generations as the only threshold. The maximum number of 5000 generations for most of the demonstrations was chosen based on computational feasibility and the simulation evaluation budget. For a simulation time of approximately 60–120 seconds per evaluation, we targeted a total runtime of one week for practical applications. This generation limit is halved to 2500 for the sheet demonstrations in Fig. S12B and Fig. S13B due to the severe computational times. In order to show that the framework continuously improves on the designs without trapping into local optima (Fig. S10), we ran the algorithm longer for three specific cases representing different structural and material complexities (Figs. S2B, S9C, and S4E).

S1.2.C – Similarity and differences of our algorithm compared to other MAP-elites variants

Our algorithm has a similar flow at the high-level to the MA-ES in terms of the decision making on exploration and exploitation, and guiding the solution candidate mutation with a hybrid approach. However, the mechanism for the guidance is drastically different. We are employing surrogate model instead of ES to assist MAP-elites with mutating promising solution candidates, where the candidates are evaluated at the simulation environment only if certain quality and novelty criteria are satisfied according to the predictions of the surrogate model. This sub-module of the algorithm is similar to the Model-Based Quality-Diversity[42] (M-QD) with regards to online training of the surrogate model and how surrogate model is employed, though M-QD employs a novelty search and local competition (NSLC) algorithm[43] as the QD search approach. Similar to our algorithm, both DA-QD and DSA-ME employs MAP-elites integrated with NN as online-trained surrogate models, however in these approaches, MAP-elites evaluates the potential solution candidates directly on the surrogate model instead of

simulation environment. This approach requires relying on a surrogate model that performs predictions at an acceptable accuracy level after a certain number of iterations, so that MAP-elites can run directly on the surrogate model. However, in the case of stimuli-responsive soft materials, we have observed sub-par accuracies for surrogate models trained in non-simplified, enormous design spaces, which impedes implementation of DSA-ME and DA-QD due to performance issues.

In addition, all of these methods (MA-ES, M-QD, DA-QD, and DSA-ME) represented the parameters with direct encoding. In our framework, we have utilized CPPN, in which the representations are learned indirectly through the mutations over iterations, for the representation of the structural design parameters. DDE-elites and PoMS combines MAP-elites with unsupervised representation learning methods to scale MAP-elites to higher-dimensional problems. They utilize generative models of Variational Autoencoder (VAE) and Autoencoder (AE), respectively. While the learning process of these models is drastically different than CPPNs, evolution of the CPPN changes the representation of the parameters. So, these methods have a somewhat slight resemblance to proposed algorithm in this study only for the representation of the parameters.

S1.3 – Benchmarking compared to selected MAP-elites variants

To assess the performance of our algorithm with respect to the pure MAP-elites and other MAP-elites variants, we conducted a comparison case based on the shape-morphing demonstration shown in Fig. 2A. Since the performance benefits of MAP-elites compared to other algorithms, including evolutionary algorithm, novel search, and CMA-ES, is well studied in literature[20,29], we specifically focused our benchmarking to pure MAP-elites, MAP-elites variants and a naïve random algorithm. Among a wide range of MAP-elites variants, we selected the DSA-ME as this algorithm trains a surrogate model in an online manner, similar to our approach, yet employing it as direct evaluation tool for the altered designs of the selected elites, as explained before in SI S1.2. In addition to this, we also implemented a MAP-elites with an offline trained surrogate model replacing the simulation environment. This variant

is inspired from a wide range of works in stimuli-responsive soft materials that employs surrogate models to replace computationally costly simulation tools[44-49]. This subsection outlines the comparison of our algorithm with respect to other algorithms and details of their implementation, as well as, the detailed explanation of the calculation of performance metrics.

S1.3.A – Implementation of the benchmarking algorithms

We benchmarked our algorithm with respect to 4 other algorithms of random, pure MAP-elites[20], DSA-ME[25] and MAP-elites with an offline trained surrogate model. All of the algorithms are run for 5000 iterations with 50 evaluations in each iteration, resulting in 2.5e5 total evaluation number. All of the algorithms implemented with the same representation method that is utilized in our work to prevent any potential bias stemming from the differences in parameter representations.

Random search

Given the vast design space, acquiring globally optimal ground truth data for demonstrations is unfeasible. Nonetheless, obtaining a rough reference point for performance is valuable. To establish this baseline for comparison with other algorithms, we employed the random algorithm.

The random naïve baseline conducts a random search within the design space utilizing the parameter representations in our algorithm. This approach generates 50 design candidates by stochastically establishing a CPPN for structural configuration and assigning the parameters for material programming for each design. The random generation of designs mirrors the initialization process of our algorithm within the MAP-elites framework. The algorithm iterates through this procedure for 5000 iterations, resulting in 2.5e5 evaluations. For further details and the exact algorithmic flow, please refer to our code within the “RandomOpt” class in the "algorithm.py" file.

MAP-elites

We adopt pure MAP-elites[20] as an additional baseline, referencing its established benchmarking against a diverse range of algorithms designed for vast search spaces. The implementation of MAP-elites within our study closely resembles the algorithmic flow of our own approach, albeit without the surrogate model submodule.

In our implementation of MAP-elites, all modified design candidates undergo direct evaluation within the simulation environment, without a selection process based on predictions from a surrogate model. This implies that in Fig. S4A, the algorithm's flow directly proceeds to simulation evaluations subsequent to design alterations, bypassing the prediction and selection steps that would rely on a surrogate model. The rest of the implementation follows the same procedure as explained in SI S1.2B. For further details and the exact algorithmic flow, please refer to our code within the “MAP_Elites” class in the "algorithm.py" file.

DSA-ME

We aimed to incorporate a MAP-elites variant combined with an online-trained surrogate model, similar to our algorithm, into the benchmarking process. Among the available options of DA-QD[24] and DSA-ME[25] algorithms, both integrating neural networks (NN) as online-trained surrogate models with MAP-elites, we opted for DSA-ME. This choice was made due to its broader applicability across various optimization problems compared to the more specialized skill learning aspect found in reinforcement learning domains of DA-QD. As detailed in SI S1.2A, DSA-ME utilizes an online-trained surrogate model similar to our algorithm. However, a notable distinction lies in how the solution candidates are handled within the MAP-elites algorithm flow. In DSA-ME, these candidates undergo direct evaluation within the surrogate model, differing from our approach.

DSA-ME is implemented following the exact algorithm flow as reported by Y. Zhang et al. [25]. To initialize the surrogate model, we utilized 2.5e5 simulation evaluation results from the “random” algorithm run. This initial dataset was used to train the surrogate model of Neural

Network (NN) over 1000 epochs. Subsequently, the surrogate model is incorporated within the initial inner loop iteration of DSA-ME. The inner loop iterates 5 times, altering the designs of 50 elites for each iteration, and updates the MAP-elites surrogate archive. Following every five inner loop cycles, the 50 top elites of the MAP-elites surrogate archive are evaluated in the simulation environment. This process updates both the ground truth MAP-elites archive and the dataset concurrently. Then, the surrogate model is trained for 20 epochs using the updated dataset. This integrated inner and outer loop combination is repeated for 5000 iterations, resulting in 2.5e5 new simulation evaluations alongside 1.25e6 surrogate model predictions. The NN architecture and the design alteration operations are exactly the same as the ones employed in our developed data-driven design algorithm to prevent any potential biases (see SI S1.2B for details). For further details and the exact algorithmic flow, please refer to our code within the "DSA_ME" class in the "algorithm.py" file.

MAP-elites with an offline trained surrogate model

In optimization problems involving computationally costly simulation evaluations, a common practice is the use of surrogate models trained with prior data to replace these simulations. This approach is widely adopted across various optimization methods, leading to significant acceleration in the operation times of optimizers. This acceleration in simulation times holds particular promise in the field of stimuli-responsive soft materials, where a single evaluation can span from minutes to hours. Given these advantages, several studies in the design of stimuli-responsive soft materials have employed similar approaches, utilizing offline trained neural networks[44-49] as surrogate models. To facilitate a comprehensive comparison of our algorithm with these works, we aim to incorporate a MAP-elites variant featuring an offline trained surrogate model that replaces the simulation environment into our benchmarking.

MAP-elites with an offline trained surrogate model are implemented similarly to our pure MAP-elites implementation outlined previously. While retaining the same algorithmic flow, the simulation model is substituted with a Neural Network (NN) surrogate model. To initialize the NN, we utilize the dataset consisting of 2.5e5 simulation evaluation results

obtained from the "random" algorithm run (same dataset employed for DSA-ME implementation). This dataset is used to train the NN for 1000 epochs, mirroring the surrogate model initialization process of DSA-ME. Subsequently, the pure MAP-elites algorithm is initialized, with all evaluations of design candidates conducted on this NN model. The NN architecture and design alteration operations remain identical to those employed in our developed data-driven design algorithm to mitigate potential biases (see SI S1.2B for details). For further insights and the exact algorithmic flow, please refer to our code within the "MAP_elites_on_NNmodel" class in the "algorithm.py" file.

S1.3.B – Performance metrics for the comparison of algorithms

We quantified QD-score, global performance, reliability, precision, coverage and performance over iterations as performance metrics, as reported in the previous literature including the original MAP-elites work[20,50]. These performance metrics are originally defined for maximizing the objective functions. However, the average RMSE calculation for shape-matching demonstrations (SI S5.1) aims for minimizing the objective function. Therefore, to be compatible with the definitions of these performance metrics, we took the inverse of the calculated average RMSE values. Utilizing this inversed objective function, we calculate the performance metrics and provide a plot showing performance for best performing designs over evaluations of 10 different seeds. In this subsection, we explain how these performance metrics are calculated.

Performance over iterations

The highest performing design for each iteration of each run is used for the performance graph in Fig. S5A-C. The algorithms are run with 10 different seeds (5 seeds for random search) and the mean and standard deviation of the best-performing designs of all runs over iterations is drawn. The final best-performing designs (at the end of the 2.5e5 evaluations) for all the runs are shown in Fig S5D.

QD-score

The QD-score is calculated by summing the performance values for each filled cell within the MAP-elites archive[50] (Fig. S5E). This metric measures the quality and diversity of the obtained archive and have been reported in the previous quality-diversity algorithm works[24,25,30,31].

Global performance

The global performance (Fig. S5F) is calculated by dividing the best-performing solution of a run with the best-performance found throughout all the algorithms[20]. This metric compares the quality of the resultant best-performing solutions across all the algorithms.

Reliability

The reliability parameter is calculated exactly as defined in the pure MAP-elites paper[20]. For this calculation, the highest-performing solution found for each cell for each run is divided by the recorded best-known performance among all runs for all algorithms for the respective cell. If a cell is not filled in any runs, it is omitted from the calculations. Also, if the specific run did not produce any solution for a cell that is normally filled by any of the other runs, it is assigned as 0. Finally, the average of this calculated value across all the cells for a given run provides the reliability score (Fig. S5G). This metric is intended for assessing the reliability of an algorithm to find high-performing solutions for each cell in the map.

Mathematical formulation:

Due to lack of an absolute ground truth, we need to define the best design archive map BM that is containing the best performing designs across all the runs for every cell, representing the ground truth. Then, the map BM can be obtained as follows,

$$BM = \max_{i \in [1,\cdots,k]} (m_i(\hat{x}))$$

where m_i is the final map for the run i, k is the total number that is counting every run of every algorithm, and $\hat{x}$ is the feature vector that is describing the coordinate of the respective cell within the feature parameters (in this case, ($\text{Voxel}_{\text{ratio}}$, $\mathbf{M}_{\theta}$)). Since we calculated the parameter

reliability for 2D shape-morphing demonstrations (Fig. 2A), the $\hat{x}$ has 2 coordinate elements in our case. If a cell in any map is empty, these cells are ignored in BM, and in further calculations. Then, we can define the global reliability $G(m)$ of a map m as follows,

$$G(m) = \frac{1}{n(BM)} \sum_{\hat{x}} \frac{m(\hat{x})}{BM(\hat{x})}$$

where $n(BM)$ defines the number of non-zero cells in BM, and $\hat{x}$ is defined within the range of the feature parameter boundary limits as $\hat{x} \in \{[x^1_{min}, \cdots, x^1_{max}; \cdots ; x^j_{min}, \cdots, x^j_{max}]\}$ with j representing total number of features (in our case, [0.6, 1] and [-π, π] for $Voxel_{ratio}$ and $\mathbf{M}_{\theta}$, respectively).

Precision

The precision parameter is relatively similar to the reliability score, but during the calculations the cells that are empty for a specific run are omitted from the calculation instead of assigning a value of 0. This metric measures the trust on an algorithm by evaluating the quality of the solution for the each filled cell relative to what is possible for that specific cell. Then, the average of this calculated value across all the filled cells for a given run provides the precision score (Fig. S5H).

Mathematical formulation:

The calculation of the precision $P(m)$ is similar to the global reliability with the difference of utilizing only the filled cells of map m. Then, $P(m)$ can be mathematically defined as follows:

$$P(m) = \frac{1}{n(BM)} \sum_{\hat{x}} \frac{m(\hat{x})}{BM(\hat{x})}$$

where $n(BM)$ defines the number of non-zero cells in BM, and $\hat{x}$ is defined within the range of the feature parameter boundary limits only for the filled cells. In this case, $\hat{x}$ with j total features is defined as,

$$\hat{x} \in \{[x^1_{min}, \cdots, x^1_{max}; \cdots ; x^j_{min}, \cdots, x^j_{max}] \mid m(\hat{x}) \; is \; filled\}.$$

Coverage

The coverage parameter measures how the algorithm performs in terms of the ability to fill the cells that are possible to fill. It is calculated by dividing the total cell number that is filled by a specific run to the total number of cells, assessing the ability of the algorithm to generate diverse designs (Fig. S5I).

S1.4 – Algorithm runtime of the demonstrations

Since the understanding of the algorithm runtime is crucial for analysis on scalability, identifying bottlenecks, and transparency, we provide the details on the total iteration number and evaluation number, as well as, algorithm runtimes per iteration for all the demonstrations in Table S6. The mean and standard deviation (std) values for the total time per iteration, the design alteration time per iteration, the evaluation time in simulation per iteration, the objective function calculation time per iteration, and the archive operation time per iteration are calculated for all the recorded iterations. It should be noted that the high std values and high deviation among the similar sized demonstrations is a result of the differences in the employed CPU computing node hardware within the cluster environment depending on the workload at a given time.

According to the Table S6, simulation evaluation time is the costliest step requiring the greatest time (~70%), thus constituting the main bottleneck. The cost of simulations prevents high number of evaluations in feasible times and hinders the generation of vast amounts of data required for the more data-intensive methods such as generative models, forward simulation, or inverse models utilizing on NN models. This is also the reason why we have only used our NN model for guiding the design alterations instead of replacing the simulation environment.

S2 – Simulation environment

As mentioned before in SI S1, our algorithm is developed to work with approximately 2.5e5 evaluations to design stimuli-responsive soft materials for the desired quasi-static and dynamic behaviors. Such a high volume of evaluations would be unfeasible using conventional FEA-based methods commonly employed in the field[8,10,12,13]. In addition, these methods are restricted to quasi-static simulations, greatly constraining the design scope for dynamic behaviors. To address these issues, we adopted a mass-spring lattice model coupled with magnetic forces and torques as a simulation engine for the dynamic behavior of magnetic soft materials. This approach delivers faster simulations compared to FEA-based methods while retaining the capability to capture dynamic behaviors. Hence, the computationally low-cost predictions facilitated by our simulation environment have empowered exploration within the presented design space.

The mass-spring lattice model is adopted from a version of the "Voxelyze" environment[51] presented by S. Kriegman et al. [5] is adapted for mechanical deformations and modified for enabling magnetic materials. "Voxelyze" implements a mass-spring lattice model with translational and rotational springs and is capable of efficiently simulating heterogeneous 3D soft bodies dynamically. We integrated the magnetic force and torque calculations into the simulation platform, enabling both the electromagnetic coil and permanent magnet setups. In our implementation, we simply calculate the magnetic forces and torques acting on each voxel, which are then included in the built-in definitions of total force and torque acting on each voxel within the "Voxelyze" environment. For the demonstrations conducted with the electromagnetic coil setup (Fig. S2G), the magnetic forces are assumed to be zero due to the uniform fields generated by the Helmholtz configuration of the coils. To calculate the magnetic forces and torques accurately for the utilized permanent magnet setup (Fig. S2F) in some of our demonstrations, we collected data points experimentally for the magnetic field values at various distances with respect to our pre-defined permanent magnet. These data points enabled the accurate calculation of the magnetic field gradients, thus, the magnetic force

acting on the robot body. Within the simulation environment, the values in Table S4 are used for density, Young's modulus (*E*) and magnetic remanence (M_r). The Poisson's ratio is assumed to be 0.49. The respective voxel sizes of the demonstrations can be found in Table S5. The developed simulation engine and the generated dataset for the demonstrations are available at the following link.

Validation of the developed simulation engine is performed through a fixed-end beam magnetized along its longitudinal axis via vibrating-sample magnetometer (VSM) under a uniform magnetic field of 1.6 T. The magnetic field for actuation is generated vertically with an electromagnetic coil setup providing uniform fields. Experiments were conducted for seven different beam samples (12 mm length x 1.6 mm width x 0.2 mm thickness) made of $Nd_2Fe_{14}B$ MQFP 10-8 and Ecoflex 00-30 at 2:1 ratio (Table S4) under uniform magnetic field strength of 0, 0.5, 1, 2, 3, 5, 7, 10, and 13 mT. Simulation results under the same conditions for experimentally measured values of density 2.41 g/cm3, Young's modulus (*E*) 200 kPa, and magnetization of 28.6 kA/m show a similar trend to the experiments (Fig. S20A). However, the simulation was over-predicting the deflections especially under small magnetic field strength (0-3 mT range). To improve the sim2real transfer capability, we fitted the parameters of Young's modulus (*E*) and magnetic remanence (M_r) by utilizing Bayesian optimization (BO), balancing the over-predictions.

Details of the parameter fitting procedure via BO:

We have implemented the Bayesian Optimization (BO) using the library provided in GPyOpt[52]. The search domain is structured as a continuous space within the bounds of [42 kA/m, 126 kA/m] and [100 kPa, 300 kPa] for the selected variables of magnetic remanence (M_r) and elastic modulus (*E*), respectively. The default GP model of GPyOpt library with a kernel of Matern 5/2 (matern52) is utilized. Expected improvement (EI) is set as the acquisition function with the acquisition weight of 1. The optimal values of *E* 150 kPa and M_r 58 kA/m is obtained after a 1000 iteration of the implemented BO (Table S4).

While the parameters of Young's modulus (*E*) and magnetic remanence (M_r) are important for quasi-static deflection results, the dynamic response requires the consideration of the damping related parameters. The mass-spring lattice model of the "Voxelyze" environment has internal, collision, and global damping parameters. Since there is no feasible way to measure these parameters experimentally, we utilized the experimental results of the vertical jumping behavior presented by W. Hu et al. [53] to find the fitting values. The fitting of the jumping height for this demonstration yields the values of internal, collision, and global damping to be 1, 0.01, and 0.001, respectively. After this fitting procedure, we observed both the temporal and the spatial behavior of the dynamic jump is approximately same. Hence, we concluded our fitting for these parameters, and set them to the aforementioned values for all the demonstrations.

We also conducted an analysis on the computational time of our simulation engine to predict the wall-clock time for the required total of ~2.5e5 evaluations, providing an educated guess for total run time of our algorithm. The computation time of the simulation engine for magnetic soft materials is characterized for various voxel numbers and voxel size ranges (Fig. S20B-D). The computation time of a single run of 0.5-sec dynamic simulation takes on average ~86 wall clock seconds for demonstrated beams (12 mm length x 1 mm width x 0.2 mm thickness) with 300 voxels (200 µm) on an Intel E5-1650 v4 processor. As the whole simulation engine is implemented in platform-agnostic C++, multiple simulation runs are easily parallelizable. These characterizations shows that ~2.5e5 evaluations parallelized into 50 cores would require ~5 days to complete (Table S6).

S3 – Shape complexity score

The complexity of shape-morphing stimuli-responsive structures are mostly evaluated qualitatively, hampering the objective progress in the field. To provide a quantitative way to compare the complexity of shape-morphing, we adapted a new method to measure the shape complexity score for tethered magnetically responsive soft materials, easily extendable to

other stimuli-responsive materials as well (Fig. S11). The total complexity score measures the total angular deviation along the length of a shape-morphing beam. This score is further normalized with respect to the length of the beam to make it a length invariant score (Fig. S11).

The methodology of shape complexity score calculation:

We developed a custom script in python to detect the shapes in the provided demonstration images, obtain the points along the shape profile, arrange the node points at the pre-defined interval distances, calculate the angular change of the edge vectors that are connecting the nodes, and compute the normalized shape complexity score. The open-source libraries of openCV and skimage are utilized for the image processing parts.

The script starts by resizing all the images to width of 360 pixels to prevent any potential artifacts due to image sizes. Then, they are converted to grayscale images. The simple inverse binary thresholding (with 210 set as threshold value) is applied on these obtained grayscale images, followed by Gaussian blurring with (5, 5) kernel and Otsu thresholding repeated 3 times. After the initial thresholding steps, the image is dilated once with (5, 5) kernel, blurred with Gaussian blur ((5, 5) kernel), eroded once with (5, 5) kernel, and once more blurred with Gaussian blur ((5, 5) kernel). Then, these steps of dilating and eroding combined with blurring repeated once more. Finally, Otsu thresholding is applied once, followed up with morphological opening and closing with (3, 3) kernel. These iterative steps eliminated any noise at the background or inside foreground of the shape.

The beam profile points are detected by applying skeletonization on processed images. The left most point is selected as the reference point for the profile, and sorted with respect to this reference point (this step requires pre-arrangement of the images to ensure the shape is rotated accordingly). The total pixel length of the shape is calculated through summation of the interval distances among the sorted points. The scaling value relating the pixel length to the beam length is obtained by dividing pixel length to the original shape length in mm. Edge length is set to 0.1 mm and total node number is calculated with respect to the

shape length ($total\,node\,number = \frac{shape\ length}{edge\ length}$). The profile points are smoothened via applying Savitzky Golay filter[54] with polynomial order of 1 and window size of $max(\frac{total\,node\,number}{20}, 5)$. The smoothened profile points are interpolated to obtain pixel position of the nodes along the profile. Then, the node pixel positions are smoothened via applying Savitzky Golay filter with polynomial order of 1 and window size 3. Finally, the obtained node pixel positions are converted to cartesian coordinates in millimeter via the previously obtained scaling value (Fig. S11A).

Finally, the edge vectors are established among the sorted node positions from one node to following node. Then, angular change in the vector directions is calculated starting from the reference node till the final node. The sum of these angular changes gives the shape complexity score. This value is divided by the shape length to obtain normalized shape complexity score ($SC_{normalized}$) (Fig. S11A).

The comparison of 2D shape-morphing demonstrations with the state-of-the-art:

Our comparison is confined to 2D shape-morphing of tethered magnetically responsive soft materials. We included all of the 2D shape-morphing cases in our work, as well as the pioneering works of G.Z. Lum et al. [55], S. Wu et al. [47], L. Wang et al. [56], and P. Lloyd et al. [57] (Fig. S11B-L)**.** The comparison of the calculated normalized shape complexity scores highlights the difficulty of the shapes obtained in our work (Fig. S11B). As can be seen in Table S1, the complexity score of our demonstrations are 10 to 20 times higher than the reported demonstrations in the works of S. Wu et al. [47], L. Wang et al. [56], and P. Lloyd et al. [57]. Extremely thin samples (60 µm) utilized in the work of G.Z. Lum et al. [55] facilitated easier bending and resulted in a relatively higher complexity score compared to other works, although still lagging far behind (1.1 to 2 times) with respect to our demonstrations.

S4 – State-of-the-art for design of stimuli responsive soft materials

S4.1 – Design of magnetically responsive soft materials

The challenges posed by the vast design space, coupled with computationally intensive simulations, have significantly constrained earlier works in design of magnetically responsive soft materials. The intricacies of this design space primarily stem from the multitude of variables, including the programmable magnetization directionality (magnetic profile, pre-defined vs. 1D vs. 2D vs. 3D), the dimensional complexity of the structural design in spatial space (pre-defined vs. 1D vs. 2D vs. 3D), and the potential incorporation of multi-material compositions. Given that multi-material capabilities have not yet been integrated into the forefront of magnetically responsive soft material design, our focus lies on the comparison of the existing methods in terms of magnetic profile and structural configuration, as illustrated in Fig. S19A. The increase in dimensionality along either axis leads to an exponential expansion of the design space (as elaborated in Table S3), necessitating the development and implementation of robust design optimization strategies.

Recent advancements in the fabrication and programming of magnetically responsive soft materials achieving 3D dimensional magnetic profiles and intrinsically 3D structural configurations resulted in a tremendous design space unfathomable before[8-12]. However, previous studies were not able to cope with this vast search space, thus constrained the dimensionality of the magnetic profile[46,47,56,58] or the structural configuration[55,59], resulting in a remarkably simplified design flexibility. While the majority of the design methods presented to date are confined mostly to pre-defined structures[46,47,55,59], or pre-defined magnetic profiles[58] (Fig. S19A), few attempted to address 1D structures with 1D magnetic profiles[56] or 2D structural configuration with sparsely discretized 2D magnetic profile[60] (Fig. S19A). These simplifications also limit the capabilities of demonstrations particularly to 2D planar deformations of rods, beams or interconnected segments.

Relatively easy adaptation of Evolutionary algorithms (EA) to a wide range of applications along with their capability to explore large search spaces, has made EA an appealing option for the design of magnetically-responsive soft materials. Several works employed EAs to design 2D magnetic profiles with pre-defined structures[55], 1D magnetic profiles with pre-defined structures[47], and 1D structures with 1D magnetic profiles[56], demonstrating desired simplified planar deformations of beams or rods. However, as the complexity of shape-morphing and the dimensionality of magnetic profiles and structural configurations increase, the scalability of EAs becomes a critical concern. Challenges such as premature convergence, sensitivity to hyperparameters, and the high computational burden associated with a large number of evaluations severely hinder the application of EAs in larger search spaces for more intricate design tasks. Although premature convergence can be mitigated through multiple runs with varying initializations, the computational overhead incurred by the sheer number of evaluations remains a significant obstacle.

Deep learning (DL) methods are employed to reduce the computational burden associated with expensive evaluations by leveraging neural networks to learn either a forward or inverse model[61]. Noteworthy applications showed the design of 1D magnetic profile for a pre-defined metamaterial structural configuration[46] and 2D magnetic profile for a pre-defined rod-shaped structure[59]. While DL methods excel at capturing intricate correlations between material properties and magnetic responses, their efficacy depends on the availability of high-quality data, the selection of an appropriate network architecture, and the fine-tuning of hyperparameters. These considerations severely limit generalizability of DL methods to unseen data with insufficient volume of training datasets. Moreover, the required data volume for effectively capturing the vast search space of high-dimensional design cases (e.g., 3D magnetic profile with 3D structural configurations) may be unattainable with the existing computationally costly simulation tools.

Topology Optimization (TO) has garnered significant acclaim for its ability to design structural configurations with optimal material distributions in a wide range of applications,

rendering it an appealing tool for addressing numerous challenging design problems. In the context of magnetically responsive soft materials, TO has been leveraged to design 2D structural configurations with either pre-defined[58] or sparsely discretized 2D magnetic profiles[60]. Despite the capability of TO to provide rationally optimized designs, its reliance on high number of evaluation iterations with Finite Element Analysis (FEA) or other mathematical models presents challenges. While achieving high iteration numbers may be feasible with simplified models, the assumptions inherent in these models significantly constrain the applicability of TO. For example, simplified models often assume linear material properties, neglecting the nonlinear behavior of magnetic materials. Additionally, they might assume quasi-static conditions, ignoring the dynamic responses and time-dependent effects that are critical in many applications. For instance, the TO framework presented by Zhao et al. [60] utilizes the nonlinear field theory for ideal hard-magnetic soft materials (as introduced by Zhao et al. [62]), assuming quasi-static deformations and a fixed-point at the structure configuration. This means the model does not account for dynamic behaviors or transient states that can occur in real-world applications. Such assumptions confine the applicability of the presented framework to quasi-static, and fixed shape-morphing designs, precluding structures exhibiting desirable dynamic behaviors. Furthermore, the intricate nonlinear coupling between the mechanical and magnetic properties needs fine discretization in modelling to capture the complex behavior, substantially increasing the computational cost of TO. Additionally, the convergence of TO is contingent upon the initial design guesses and hyperparameters, necessitating a plausible starting design guess for optimal algorithm performance. These challenges collectively hinder the scalability of TO across a broader range of application in magnetically responsive soft materials.

The design strategy developed in this work surpasses these existing limitations by enabling the simultaneous design of 3D magnetic programming profiles and 3D structural compositions. The inherently scalable nature of our framework promises a broader applicability beyond magnetically responsive soft materials. In addition to magnetic

programming capability and structural complexity, multi-material compositions with other stimuli-responsive or passive soft materials are also crucial for expanding the capabilities of intelligent soft materials, along with increased challenges in design strategies. In the next section, we provide an overview of state-of-the-art design frameworks and our approach for overcoming these challenges for stimuli-responsive soft materials.

S4.2 – Design of stimuli-responsive soft materials

The primary limitations in designing stimuli-responsive soft materials arise from the vast and often deceptive solution space, coupled with the computationally intensive nature of evaluating potential solutions. This vast design space emerges from the intricate coupling of 3D programmability of responsive materials and the incorporation of multi-material compositions into 3D structures. Modelling responsive materials with deformable soft bodies, highly coupled multi-physics phenomena, and nonlinear behaviors results in computationally costly simulation engines, further constraining the evaluation rate of potential solutions. Addressing these challenges necessitates design methods that effectively balance exploitation and exploration in a sample-efficient manner, while avoiding premature convergence to suboptimal solutions. Furthermore, they must scale efficiently to accommodate the increasing dimensionality and complexity resulting from the integration of multi-responsive materials and higher-resolution material programmability, as well as advancements in structural fabrication techniques[8-12,63-67].

The majority of the existing methods rely on evolutionary algorithm for searching the design space[5,68], requiring a large number of evaluations with multiple runs due to convergence to local optima and inefficient exploration. The computational cost of evaluating a high number of potential solutions limits scalability of these approaches for stimuli-responsive soft materials. Recent methods have incorporated various Neural Network architectures as surrogate models[44] to replace the simulation engines, thereby overcoming the computational burden of the costly evaluations. However, these methods are primarily limited to responsive materials with scalar, 1D programmability and 2D or 1D structural

designs, dealing with a drastically smaller design space[5,44] (Fig. S19B). The scaling of these methods to higher dimensional programmability and structural designs with hundreds of order higher search spaces is highly questionable due to the required vast dataset of simulation evaluations to train surrogate models at reasonable accuracies to replace simulation engines.

While the potential of more sophisticated NN architectures, such as Generative Adversarial Network[69] (GAN), to generate high performance solutions within relatively larger design spaces compared to the aforementioned methods is shown[45,48,70,71], it is currently limited to 2-dimensional structures with single passive (inert) soft materials. Extending GANs to multi-material, 3D structures with 3D programmability would require substantially more training data than 1.7e7 data points, as deployed in the previous work[70]. Additionally, the NN architectures should be further studied to effectively capture the coupled interaction between 3D programmable material response to stimuli and 3D inherent structural design.

Topology Optimization (TO) is a well-established and powerful strategy for structural design with optimal material distributions. Despite its wide-range of applications in coupled multi-physics design problems[72] only a handful of works address the design of stimuli-responsive materials[73-75], which are mostly limited to scalar (1 dimensional) material programmability[73], and beam[74] or pre-defined structural configurations[75,76]. While a recent report showed the design of sparsely discretized 2D programmable (8 distinct magnetization directions) and 2D structural configurations for magnetically-responsive soft materials[60], demonstrations were limited to quasi-static and fixed-point structures due to the assumptions of the utilized physics model[62]. The requirements of complicated and difficult gradient computations for sensitivity analysis at each design refinement for highly nonlinear, coupled and discontinuous physics, such as a soft robot with large-deformations under external stimuli with contact, leads to computationally expensive physical simulations and design iterations. Despite these limitations, the recent developments in the field of TO to achieve faster computational times, dynamic capabilities, and material nonlinearities by combining TO with

differentiable physics[77] and machine learning methods[17,78,79], show promises for the applicability of TO for stimuli-responsive soft materials field in the future.

Stochastic gradient descent (SGD) and Bayesian optimization (BO) are other widely accepted methods for design problems. SGD relies on gradient information and tend to have slower convergence rates with the possibility of getting stuck in local optimal points and high sensitivity to the initial starting points in highly non-convex search spaces. Moreover, computational cost of obtaining gradient information in stimuli-responsive materials can be extremely high especially with increased parameter number. These challenges render SGD not a viable option for the design of stimuli-responsive soft materials as also shown comparably in previous reports[5,55] with respect to EA method. On the other hand, BO operates based on iteratively updating a surrogate model, mostly Gaussian Processes, and performing acquisition function optimization. The computational complexity of building the surrogate model makes BO impractical for high dimensional (>20 parameters) search spaces[80,81]. Despite the wide-spread use of SGD and BO, both greatly suffers from the curse of dimensionality and has scalability issues for the design of stimuli-responsive soft materials.

Overall, the prominent methods[5,44,49,70,76], highlighted in Fig. S19B, attempt to design stimuli-responsive soft materials by heavily simplifying the design space. On the other hand, the other works dealing with relatively large design spaces (> 1e100) are limited to passive materials[5,49,70] or materials with severely limited programmability[44,76]. The capability to design 3D inherent structures, local domain programmability up to 3D orientations, and multi-material compositions with other stimuli-responsive or passive soft materials within a large design space (~1e1000) underscores the robustness and broad applicability of our design strategy compared to prominent existing methods (Fig. S19B).

S5 – Performance measurements

The performances of the designs are measured through the pre-defined performance objective functions. While the average positional root mean square error (RMSE) is utilized for

all the shape-matching demonstrations, the behavioral demonstrations have different performance objective functions. This section provides the details of the utilized performance objective functions and the shape-definitions of the shape-matching demonstrations.

S5.1 – Performance objective functions

Shape-matching demonstrations, minimizing positional error

The performance objective function for the shape-matching demonstrations (Fig. 1G, Fig. 2, and Fig. S9) is defined as the sum of RMSE between the desired shape and the simulation result for all voxel positions averaged by the total voxel number. The objective function, *f*, is calculated as follow:

$$f = \frac{1}{N}\sum_{i=1}^{N}\sqrt{\left(x_d^i - x_s^i\right)^2 + \left(y_d^i - y_s^i\right)^2 + \left(z_d^i - z_s^i\right)^2},$$

where *N* is the number of voxels, and the (x_d^i, y_d^i, z_d^i) and (x_s^i, y_s^i, z_s^i) are the ith voxel coordinates for the desired shape and simulation results, respectively. The algorithm aims to minimize this function over the iterations. For 2D shape-matching demonstrations, a simplified version with only (x_d^i, z_d^i) and (x_s^i, z_s^i) is utilized.

Maximizing the turn number of a beam around longitudinal axis

The performance objective function is defined with the total orientation change around the longitudinal axis divided by 1 turn periodicity of 360 degrees (Fig. S12A). The objective function, *f*, for turn number is calculated as follows:

$$f = \frac{1}{2\pi}\sum_{i=1}^{K}(Avg.Ori.(i+1) - Avg.Ori.(i)),$$

where i is the slice number, *K* is the total slice number in longitudinal axis directions, $Avg.Ori.(i)$ represents the average orientation of voxel slices in transverse axis (along the width of the beam, y-axis) for i^{th} slice, which is calculated as follows:

$$Avg.Ori.(i) = \frac{1}{L}\sum_{j=1}^{L}\theta(i,j),$$

where i is the slice number, L is the total voxel number in transverse axis, j is the voxel coordinate in transverse axis (y-axis), $\theta(i,j)$ is orientation of the respective voxel in radians. Single arrays of voxels in the transverse axis are defined as slices, separating the voxels along the longitudinal axis.

Maximizing the height of the center point of a sheet

The average height of the voxels at the center is defined as the performance objective function (Fig. S12B). The set of voxel indices to maximize in z position are defined as $V = ((i, j) \mid 23 < i < 28, 23 < j < 28)$ for a 2D grid of voxels with dimensions of [50, 50]. Then, the objective function, f, is calculated as follows:

$$f = \frac{1}{N}\sum_V z(i,j)_s,$$

where N is the total number of voxels, V is the set of voxel indices, $z(i,j)_s$ is the respective voxel position in z axis. It is worthwhile to mention the parameter N can vary depending on the structural design as some of the voxels defined in set V could be empty.

Minimizing the bounding sphere volume of a sheet

The performance objective function is defined as the radius of the smallest sphere that is enclosing all the voxels of the sheet (Fig. 3A). The smallest bounding sphere is calculated utilizing the "get_bounding_ball()" function of an open-source library "miniball 1.2.0" [82]. The function is provided with the voxel positions of the sheet and the precision parameter "epsilon" is set to 1e-9.

Vertical jumping behavior

The vertical jumping behavior is demonstrated for 2D beam, sheet and multi-material as well as, 3D multi-material structures (Fig. 3D, Fig. 4C, Fig. S13B, and Fig. S15). The performance objective function is defined in terms of the defined rewards and penalties for all the aforementioned demonstrations besides 2D beam jumper inspired from reinforcement learning as follows:

$$f = rewards - penalties.$$

The rewards are calculated as follows:

$$rewards = 10 * time_{jumping},$$

where $time_{jumping}$ indicates the total time spent on air by the robot, and the penalties are calculated as follows:

$$penalties = \begin{cases} 0, & time_{jumping} > 0 \\ Voxels_{Contact} + 900, & \text{otherwise} \end{cases},$$

where $Voxels_{Contact}$ indicates the minimum number of voxels contacting the surface at any time during the evaluation of the robot behavior, and an additional value of 900 is added to the penalty if the $time_{jumping}$ is not greater than 0, implying the robot could not jump.

For the demonstrations of 2D beam jumper, the maximum center of mass height (COM_z) in mm is added to the rewards as follows:

$$rewards = 10 * time_{jumping} + \max(COM_z),$$

and final center of mass position in x and y axes (COM_x^{final}, COM_y^{final}) in mm are added to the penalty function as follows:

$$penalties = \left(COM_x^{final} + COM_y^{final}\right) + \begin{cases} 0, & time_{jumping} > 0 \\ Voxels_{Contact} + 900, & \text{otherwise} \end{cases}.$$

Directional jumping behavior

The performance objective function of the directional jumping behavior with 3D multi-material structures (Fig. 4D) calculated in a similar manner with the defined rewards and penalties. While the penalty function is same as the vertical jumping, the rewards have additional terms. The rewards are calculated as follows:

$$rewards = 100 * time_{jumping} + COM_x^{disp.} + \max(z_{min}),$$

where $time_{jumping}$ indicates the total time spent on air by the robot, $COM_x^{disp.}$ is the displacement of the center of mass in x direction, and the $\max(z_{min})$ denotes the maximum of the minimum voxel height within the robot during the jumping behavior. The penalty function is same as the vertical jumping cases, and calculated as follows:

$$penalties = \begin{cases} 0, & time_{jumping} > 0 \\ Voxels_{Contact} + 900, & \text{otherwise} \end{cases},$$

where $Voxels_{Contact}$ indicates the minimum number of voxels contacting the surface at any time during the evaluation of the robot behavior, and an additional value of 900 is added to the penalty if the $time_{jumping}$ is not greater than 0, implying the robot could not jump.

Traversing robot behavior

Traversing 3D multi-material soft robot (Fig. 4E) is achieved by maximizing the center of mass displacement in x direction ($COM_x^{disp.}$) (in mm), and the performance objective function is calculated as follows:

$$f = COM_x^{disp.}.$$

Maximizing force generation of a multi-material and 3D structure

The total force in z direction for a slice of predefined voxels at the bottom of the structure is defined as the performance objective function (Fig. S17). The set of voxel indices are defined as $V = [(i, j, k) \mid 0 < i < 8, 0 < j < 4, k=1]$ for a 3D arrangement of voxels with dimensions of [7, 3, 30]. Then, the objective function, f, is calculated as follows:

$$f = \sum_V F_z(i,j,k),$$

where $F_z(i,j,k)$ is the force in z direction for the respective voxel, and V is the set of voxel indices.

Configurable traversing and directional jumping behaviors

The performance objective function is defined as the functions of rewards and penalties for traversing and directional jumping behaviors (Fig. S18). Since this desired configurable behavior presents a highly challenging design problem, the objective function is relatively complicated compared to previous demonstrations. The objective function, f, is defined as follows:

$$f = rewards - penalties,$$

$$rewards = reward_{jumping} + reward_{traversing},$$

$$penalties = penalty_{jumping} + penalty_{traversing} + penalty_{difference},$$

where the rewards are the sum of $reward_{traversing}$ and $reward_{jumping},$ and penalties are the sum of $penalty_{traversing}$, $penalty_{jumping}$ and $penalty_{difference}$, respectively.

The terms of reward and the penalty for directional jumping part is similar to single directional jumping behavior and calculated as follows:

$$reward_{jumping} = 100 * time_{jumping} + COM_x^{disp.} + \max(z_{min}),$$

$$penalty_{jumping} = \begin{cases} 0, & time_{jumping} > 0 \\ Voxels_{Contact} + 200, & \text{otherwise} \end{cases},$$

where $time_{jumping}$ indicates the total time spent on air by the robot, $COM_x^{disp.}$ is the displacement of the center of mass in x direction, the $\max(z_{min})$ denotes the maximum of the minimum voxel height within the robot, $Voxels_{Contact}$ indicates the minimum number of voxels contacting the surface at any time during the evaluation of the robot behavior, and an additional value of 200 is added to the penalty if the $time_{jumping}$ is not greater than 0, implying the robot could not jump.

The rewards and the penalty terms for traversing part is more involved compared to the directional jumping part. The reward for traversing behavior is calculated as follows:

$$reward_{traversing} = \sum_{steps} COM_x^{disp.}(step) * step^2,$$

where $COM_x^{disp.}(step)$ represent the center of mass displacement for a given step, and step indicates the magnetic field cycle number. The same pre-defined magnetic field is repeated five times, resulting in a total of 5 steps. The presented $reward_{travesing}$ function is defined as the sum of the displacement of the center of mass for each step multiplied by the square of the step number. This reward encourages traversal behaviors where the displacement of the center of mass increases progressively with each step, as indicated by the quadratic term. Overall, the expression incentivizes traversals where the center of mass moves consistently and sufficiently, with higher rewards for greater displacement and step progression. The penalty for traversing behavior is calculated based on whether certain conditions related to the displacement of the center of mass, as well as, jumping occurrences and calculated as follows:

$$penalty_{traversing} = 3 * std\left(\overline{\boldsymbol{COM_x^{disp.}}}\right) + \begin{cases} 20, & time_{jumping} > 0 \\ 0, & \text{otherwise} \end{cases}$$

where $\overline{\boldsymbol{COM_x^{disp.}}}$ denotes the vector of center of mass displacements for all steps, $time_{jumping}$ indicates the total time spent on air by the robot, and std is the standard deviation function. The standard deviation captures the variability or spread of the displacement values across all steps during traversing behavior. Overall, the traversing penalty increases if jumping occurs, or there is high variability in the displacement across steps.

Finally, a penalty for difference between the distances covered by the traversing and the directional jumping cases is calculated as follows:

$$penalty_{difference} = \sqrt{\left(COM_{x,jumping}^{disp.} - COM_{x,traversing}^{disp.}\right)^2},$$

where $COM_{x,jumping}^{disp.}$ represents the center of mass displacement for directional jumping behavior, and $COM_{x,traversing}^{disp.}$ indicates the total center of mass displacement for traversing behavior. This penalty calculation determines how much the jumping behavior's final position differs from the traversing behavior, potentially penalizing the deviations in an increasing rate.

S5.2 – The mathematical representations of the desired shapes for shape-matching demonstrations

This subsection provides the mathematical representations for the shape-morphing demonstrations in Fig. 2 and Fig. S9. These representations are mostly defined with parametric and piecewise functions without the exact scaling. The shapes are shifted in x and z axis to start from origin and scaled with respect to the beam lengths. After the processing of these shapes, desired voxel locations are obtained at equidistant positions. The details can be found at the "generateDesiredShape()" function provided in our code (https://github.com/AlpKaracakol/data_driven_magnetic_soft_material_design).

S5.2.1 – 2D shapes

Triangular wave (Fig. 1F, G)

This shape (Fig. 1F, G), named as "triangleWave" in our code, is obtained by defining 4 points in space and the line segments between these points. Let $P_i = (x_i, y_i, z_i)$ denote the coordinates of the ith point. Then, the points are defined as $P_0 = (0, 0, 0)$, $P_1 = (1, 0, -1)$, $P_2 = (3, 0, 1)$, and $P_3 = (4, 0, 0)$. By representing the lines between these points, the points on this shape can be obtained as follows:

$$P_t(x(t), y(t), z(t)) = \begin{cases} 0 \leq t \leq 0.25\colon\ (4t,\ 0,\ -4t) \\ 0.25 \leq t \leq 0.75\colon\ (1 + 4(t - 0.25),\ 0,\ -1 + 4(t - 0.25)) \\ 0.75 \leq t \leq 1\colon (3 + 4(t - 0.75),\ 0,\ 1 - 4(t - 0.75)) \end{cases}.$$

Varying sinusoidal signal (Fig. 2A)

This shape (Fig. 2A) named as "Heartbeat" in our code, obtained by varying the amplitude, and the frequency of a sinusoidal wave. For this, we defined sampling range of 1.5 period of sinus wave (0, 3π) with the frequency range of (1.5, 1) and amplitude range of (0.4, 2). Any point on this shape can be represented mathematically for t in the range of [0, 3π] as follows:

$$P_t\big(x(t), y(t), z(t)\big) = \big(t, 0, A(t) * \sin\big(t * F(t)\big)\big),$$

where t ranges from 0 to 3π, $A(t) = 0.17t + 0.4$ representing the variation of the amplitude with respect to t, and $F(t) = -0.053t + 1.5$ representing the variation of the frequency with respect to t.

Square signal (Fig. 2B)

For the shape of Fig. 2B, we inspired from square wave signals. This shape named as "Stepv2" in our code, can be represented with a piecewise function as follows:

$$P_t(x(t), y(t), z(t)) = \begin{cases} 0 \leq t < 2.4\colon\ (t, 0, 0) \\ 2.4 \leq t < 4.8\colon\ (\ t, 0, 2.4) \\ 4.8 \leq t \leq 7.2\colon\ (t, 0,0) \end{cases}.$$

The overall function shape can be also represented in terms of unit step function as well. In that case, the equation becomes $P_t(x(t), y(t), z(t)) = (t, 0, 2.4 * (H(t - 2.4) - H(t - 4.8)))$ where $H(t)$ is the unit step function and t is defined in the range of [0, 7.2].

Diamond sign (Fig. 2C)

Similar to triangular wave shape, the diamond shape (named as "diamond" in our code) is obtained by defining 5 points in space. The points are connected with each other by line segments sequentially to obtain the final shape. The defined points are P_0 = (0, 0, 0), P_1 = (1, 0, 1), P_2 = (2, 0, 0), P_3 = (1, 0, -1), and P_4 = (0.5, 0, -0.5). By defining each line segment equation with appropriate conditions, a piecewise equation can be obtained as follows:

$$P_t(x(t), y(t), z(t)) = \begin{cases} 0 \leq t \leq 1\colon\ (0, 0, 0) + t(1, 0, 1) \\ 1 \leq t \leq 2\colon\ (1, 0, 1) + (t - 1)(1, 0, -1) \\ 2 \leq t \leq 3\colon (2, 0, 0) + (t - 2)(-1, 0, -1) \\ 3 \leq t \leq 4\colon (1, 0\ , -1) + (t - 3)(-0.5\ ,0\ ,0.5) \end{cases}.$$

This equation represents the entire path of the desired shape from point P_0 to point P_4 as a single function of the parameter t.

Step signal (Fig. S9A)

This desired shape (Fig. S9A), named as "Stepv1" in our code, is inspired from the unit step function. Similar to "Stepv2" (Fig. 2B), we can represent (Fig. S9A) in terms of unit step

function H(t) as $P_t(x(t), y(t), z(t)) = (t, 0, 4 * H(t-4))$ for t in the range of [0, 8]. The piecewise function representation of this function can be written as follows:

$$P_t(x(t), y(t), z(t)) = \begin{cases} 0 \le t < 4: & (t, 0, 0) \\ 4 \le t < 8: & (t, 0, 4) \end{cases}.$$

Fibonacci spiral (Fig. S9B)

This shape, named as "Fibonacci" in our code, is inspired from the Fibonacci spiral. The spiral pattern can be obtained by the following parametric equation:

$$P_t\ (x(t), y(t), z(t)) = ((a + b * e^t) * \cos{(t)}, 0, -(a + b * e^t) * \sin{(t)}\ ),$$

where t is in the range of [0, 2π], $a = 10$ and $b = 0.3063489$ are the parameters controlling the starting radius of the spiral and the spiral expansion rate, respectively. The obtained shape is then mirrored around z-axis and x-axis. Then, the starting point is shifted to the origin to get exact desired shape of Fig. S9B.

S5.2.1 – 3D Shapes

3D spherical spiral (Fig. 2D)

This shape (Fig. 2D) named as "SeaShell" in our code, is inspired from the sea shells with varying 3D spherical spiral shapes. The defined shape exhibits a helix with varying radius, and can be represented parametrically as follows:

$$P_t\big(x(t), y(t), z(t)\big) = (b * t, a(t) * \sin(\mathrm{t}), a(t) * \cos(t)),$$

where $a(t) = f(t) * (3.8\pi - t)$, $f(t) = 1.5 - 0.75 * (t - \pi)/1.8\pi$, and $b = 5$ for a range of t in [1π, 2.8π]. The position vector can be obtained by combining these equations.

3D step with 4 sharp corners (Fig. 2E)

As a follow up to the 2D shapes inspired by unit step function and square wave signals (Fig. S XA, Fig. 2B), we come up with 3D versions with 4 sharp cornered turns in 3D. The 3D step shape (Fig. 2E), named as "step3D" in our code, is defined as line segments that is connecting

the pre-defined points in space. For this shape, the selected points are $P_0 = (0, 0, 0)$, $P_1 = (0.5, 0, 0)$, $P_2 = (0.5, 1, 0)$, $P_3 = (1.5, 1, 0)$, $P_4 = (1.5, 1, 1)$ and $P_5 = (1.5, 0, 1)$. The piecewise equation describing the line segments among these points can be represented as follows:

$$P_t(x(t), y(t), z(t)) = \begin{cases} 0 \leq t \leq 1: \ (0.5t, 0, 0) \\ 1 \leq t \leq 2: \ (0.5, t-1, 0) \\ 2 \leq t \leq 3: (0.5 + (t-2), 1, 0) \\ 3 \leq t \leq 4: (1.5, 1, t-3) \\ 4 \leq t \leq 5: (1.5, -t+5, 1) \end{cases}.$$

Helix (Fig. 7C)

The shape named as “Helix” (Fig. 7C) is a simplified version of the shape in Fig. 2D. The defined shape exhibits a single turn helix with constant radius, and can be represented parametrically as follows:

$$P_t\big(x(t), y(t), z(t)\big) = (b * t, a * \sin(\mathrm{t}), a * \cos(t)),$$

where a and b constants are equal to 5 ($a = b = 5$) and the range of t is in [0, 2π]. The position vector for points along the helix shape can be obtained by the parameter t.

3D step with 3 sharp corners (Fig. 7D)

In addition to 3D step shape (Fig. 2E) (“step3D” in code), we define another version with 3 sharp corners as well (Fig. 7D), named “step3Dv2” in code. Similar to the Fig. 2E, the desired shape is defined by the line segments among the selected points of $P_0 = (0, 0, 0)$, $P_1 = (1, 0, 0)$, $P_2 = (1, 1, 0)$, $P_3 = (1, 1, -1)$, and $P_4 = (2, 1, -1)$. The piecewise equation describing the line segments among these points can be represented as follows:

$$P_t(x(t), y(t), z(t)) = \begin{cases} 0 \leq t \leq 1: \ (t, 0, 0) \\ 1 \leq t \leq 2: \ (1, t-1, 0) \\ 2 \leq t \leq 3: \ (1, 1, 2-t) \\ 3 \leq t \leq 4: \ (t-2, 1, -1) \end{cases}.$$

S6 – Design space calculation

The design space (Ω) is defined as the total number of possibilities enabled by combined variations in structural design (morphology) and material programmability (magnetization direction in this work). Morphology is represented by presence or absence of individual voxels along with the type of selected material and magnetization directions are encoded for individual segments, composed of desired number of voxels. According to the definitions of voxels and segments, the design space (Ω) can be roughly calculated as $\Omega = (V)^n\,(S)^m$, where n and m define the number of voxels and segments, respectively. The *V* and *S* is the number of distinct possibilities for voxel material choices and segment magnetization directions, respectively.

The value of *V* is calculated as the total number of material choices plus the addition of empty voxel (*V* = number of materials + 1). The value *S* is the distinct possible magnetic programming directions and has a continuous parameter space for magnetization $S = [\mathbf{M}_\theta, \mathbf{M}_\varphi \mid 0 \le \mathbf{M}_\theta < 2\pi, 0 \le \mathbf{M}_\varphi < \pi]$. For the demonstrations fabricated via heat-assisted magnetic programming, the discretization of the magnetization directions in 1° for segments results in 360 and 64800 (=360*180) distinct possibilities for each magnetized segment in 2D and 3D, respectively. The demonstrations fabricated via voxel-based assembly has 6 distinct directions for each magnetized segment (*S*=6). Incorporating the respective values of *V*, *S*, *n*, and *m* for the demonstrations yields the design space values listed in Table S3. The Table S3 shows the resulting calculated design spaces for magnetization directions discretized in 1 degree and 5 degrees with and without the consideration of the morphology (structural design). These calculations further clarify and highlight the expansion of design space depending on the discretization of the magnetization directions and involvement of the structural design. The details of various parameters for calculating the values of *V*, *S*, *n*, and *m* can be found in Table S5.

An example of how design space is calculated:

The total design space size (Ω) can be roughly approximated by:

$$\Omega = (V)^n\ (S)^m,$$

Where:

- V: The number of material types, including the "void" option for empty voxels,
- n: The total number of voxels defining the structural design,
- S: The number of discretized magnetization directions available for each segment,
- m: The number of segments for magnetization programming.

Following these definitions, these parameters could be calculated via the help of the provided parameters in Table S5 as,

- V = number of materials + 1
- n = Voxel number in X axis * Voxel number in Y axis * Voxel number in Z axis
- S depends on the magnetic programming method
  - In heat-assisted magnetic programming,
    - For 2D demonstrations with 1° discretization, S = 360,
    - For 3D demonstrations with 1° discretization, S = 360*180 = 64800.
  - In voxel-assembly method S is restricted to 6 discrete directions in Cartesian axes, thus S = 6.
- m = Segment number in X axis * Segment number in Y axis * Segment number in Z axis

As an example, for the demonstration in Fig. 4, from the Table S5, we can obtain,

- V = 4 + 1 =5
- n = 7 * 7 * 5 = 245
- S = 6 (voxel-assembly fabrication)
- m = 7 * 7 * 5 = 245

Thus, the design space size,

- $\Omega = (5)^{245}\ (6)^{245} \approx 7.8e361$

Supplementary figures

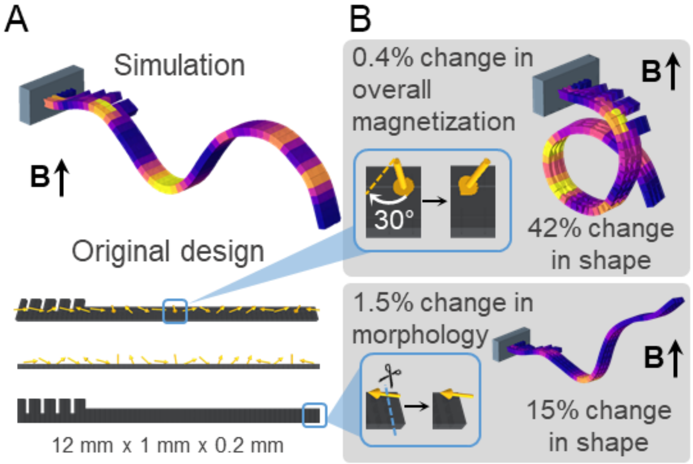

Figure S1. Marginal design alterations lead to non-intuitive dramatic changes in the shape-morphing behavior of magnetic soft materials. (A) Simulation result of a magnetic soft beam structure with distributed magnetization and non-uniform morphology deforming into a sinusoidal-like shape under a magnetic field. **(B)** Two separate marginal changes are applied to the magnetic profile and morphology of the original design to highlight the non-intuitive behavior of the magnetic soft materials. The magnetization direction of a single section is rotated by 30°, corresponding to a 0.4% change in the overall magnetic profile, leading to an average of 42% change in the resulting shape. As a separate case, the morphology of the design is changed by cutting a single voxel line laterally corresponding to a 1.5% change in overall morphology, leading to an average of 15% change in the resulting shape. Shape-morphing change ratio is calculated by the average displacement, with respect to the original design, normalized to the body length. Actuation is performed by applying a magnetic field (**B**) of 30 mT in the direction indicated by the black arrows. Magnetization directions are depicted via yellow arrows.

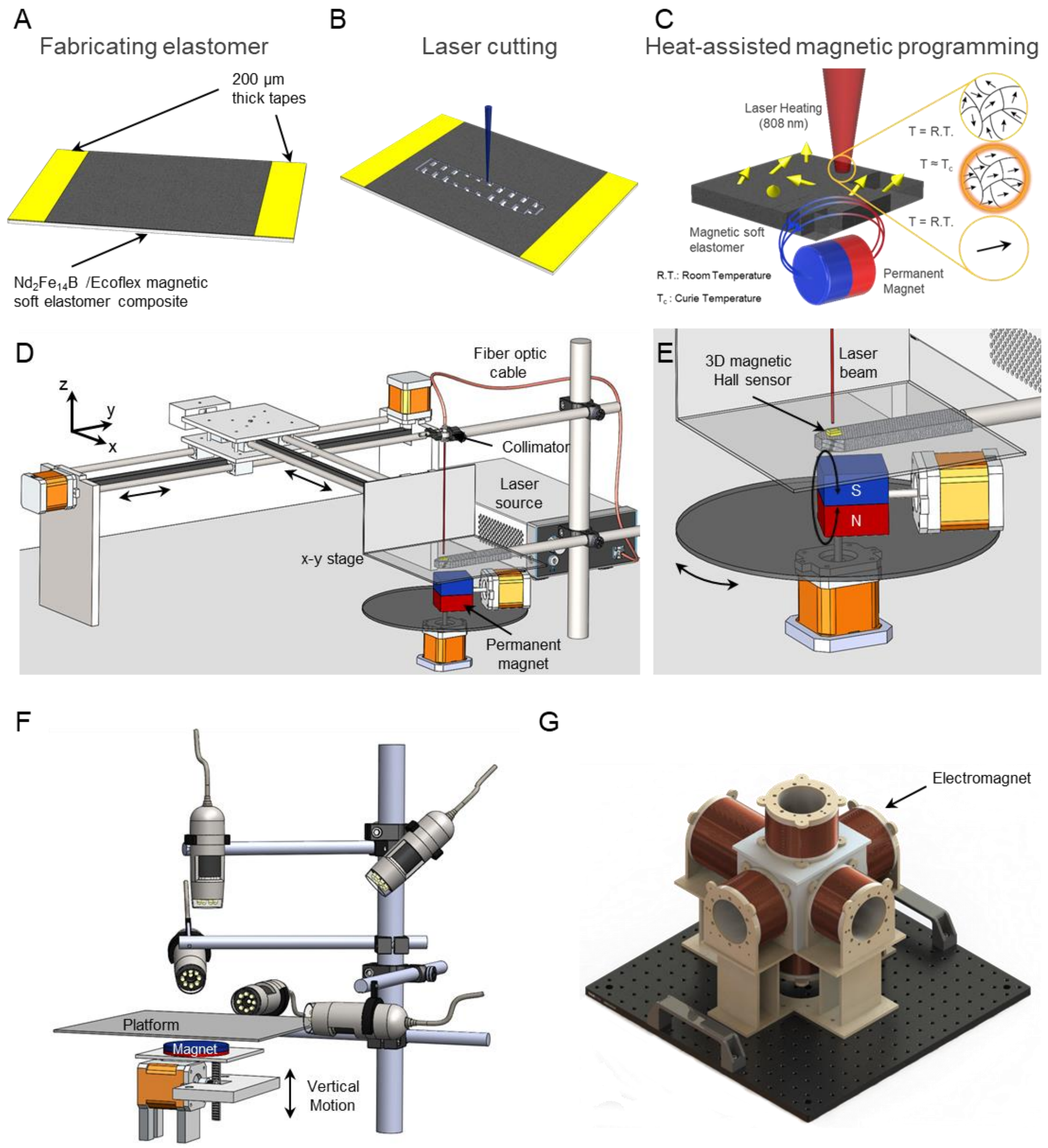

Figure S2. Fabrication, heat-assisted magnetic programming, magnetic actuation of magnetic soft elastomers. (A) $Nd_2Fe_{14}B$ /Ecoflex magnetic soft elastomer composites are prepared by mixing $Nd_2Fe_{14}B$ particles into Ecoflex at a 2:1 ($Nd_2Fe_{14}B$:Ecoflex 30) mass ratio. The mixture is cast into molds composed of tapes of 200 µm thickness adhered on a flat PMMA substrate and cured at room temperature for 4 hours. **(B)** Magnetic soft elastomers of desired morphology are cut via laser micromachining. **(C)** Designed magnetic profiles are encoded via heat-assisted magnetic programming. During the magnetization process, each segment is locally heated around the Curie temperature of the $Nd_2Fe_{14}B$ particles via a NIR

laser. The particles lose their magnetization, and their magnetization direction is reoriented by applying an external magnetic field at desired orientation during cooling. **(D, E)** The magnetic programming setup consists of a power-adjustable NIR laser with a collimator, motorized stage, 3D Hall-effect sensor, and a permanent magnet that can be rotated in 3D. **(F)** Magnetic actuation is realized by a disc-shaped permanent magnet (60 mm diameter, 20 mm thick) moved vertically via a linear stage. Cameras are fixed at various viewpoints for visual capture from isometric, top, side, and front views. **(G)** Six-coil magnetic setup in Helmholtz configuration is used for magnetic actuation of the magnetic soft elastomers under uniform magnetic fields.

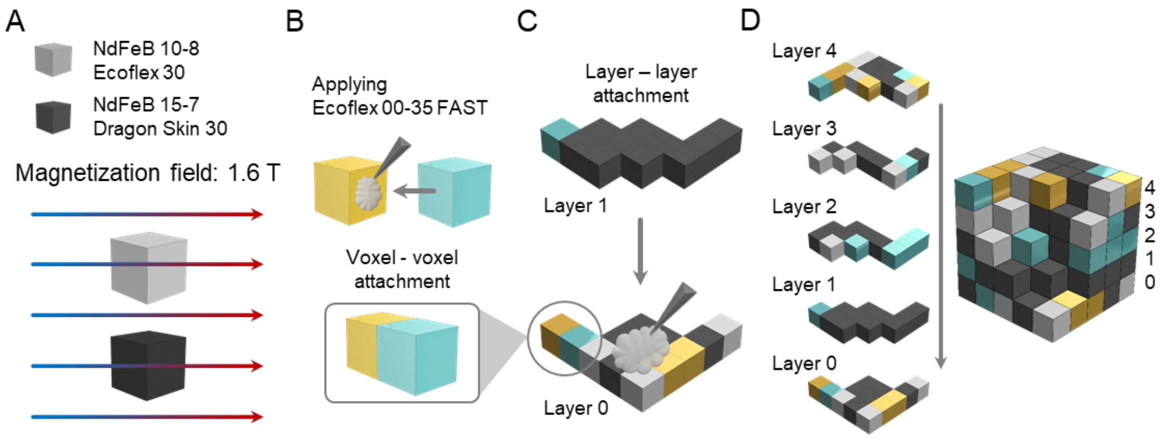

Figure S3. Fabrication and programming of multi-material and 3D structures based on voxel-assembly method[12]. (A) The magnetic materials are hard magnetized under 1.6 T prior to fabrication. **(B)** The voxels are attached to each other by applying Ecoflex 00-35 FAST, and mechanically hold together until the Ecoflex 00-35 FAST is cured. **(C, D)** The layers are fabricated individually by voxel-voxel attachments, followed by the attachment of layers to each other **(C)**. The layer-layer attachments of multi-layers results in 3D multi-material structure **(D)**.

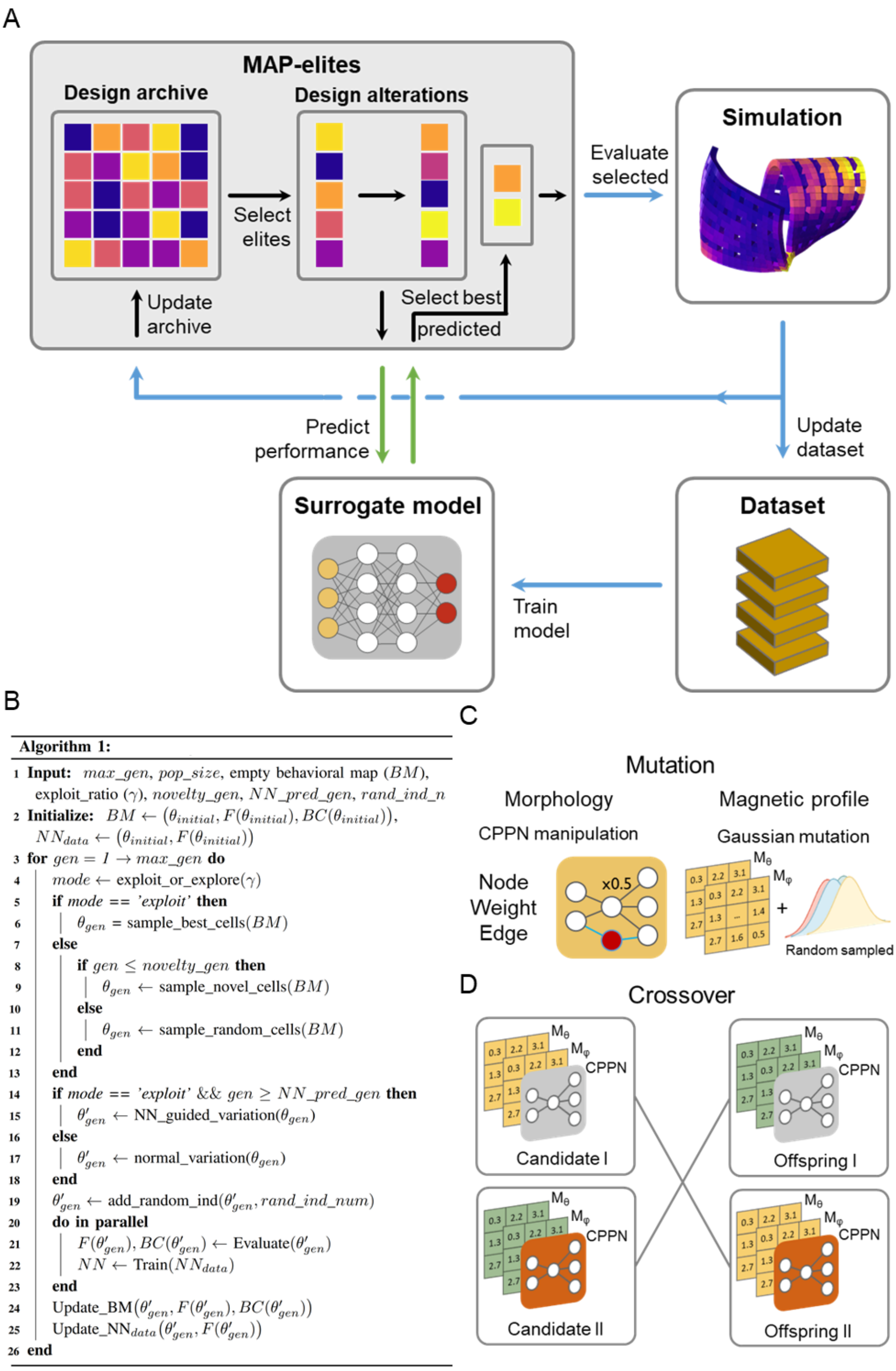

Figure S4. An overview of the developed algorithm for data-driven design of stimuli-responsive soft materials. (A) The algorithm flow of the developed data-driven design strategy for stimuli-responsive soft materials. The MAP-elites archive is initialized by randomly generated design candidates evaluated in the simulation environment. At the beginning of

each iteration, a set of elites is selected according to predefined exploitation or exploration rules. The design of the selected group is altered by mutation or crossover operations. The performances of the modified design candidates are predicted on the surrogate model to identify and select the candidates with the highest performance predictions, which are then evaluated in the simulation environment. The evaluated designs are then added to the dataset and the design archive is updated. The surrogate model is trained online with the latest dataset in parallel during the evaluation of the design candidates in the simulation environment. **(B)** The pseudo-code of the developed neural network guided MAP-elites algorithm for data-driven design of stimuli-responsive soft materials. **(C, D)** The design alteration operations of mutation and crossover. **(C)** During the candidate generation process, mutations are introduced randomly either on CPPN or magnetic profile. Mutation on CPPN randomly adds/removes a node, adds/removes an edge, or changes the weight of an existing edge. Mutation on magnetic profile (**M**) randomly chooses a segment and applies random sampled gaussian mutation. **(D)** Crossover operation is defined as the interchange between morphology and magnetic profile (**M**). Candidates chosen randomly for crossover operation exchange their magnetic profiles ($\mathbf{M}_{\theta}$ & $\mathbf{M}_{\varphi}$ parameter matrices). This operation ensures an exchange of morphology and magnetic profile among the design candidates, enhancing better performing generic morphologies and magnetic profiles to be shared among other candidates.

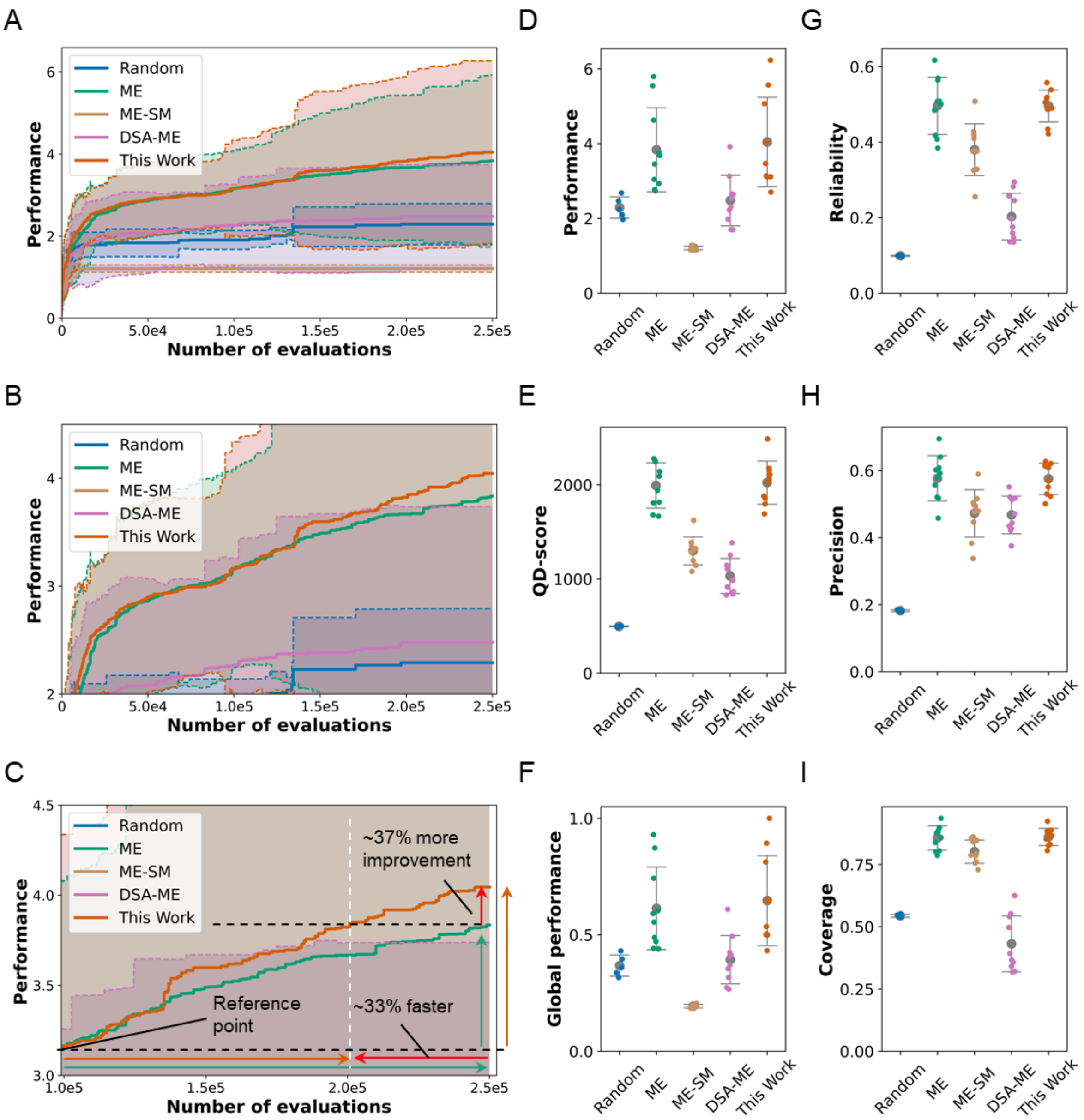

Figure S5. The comparison of our algorithm with other baseline algorithms for the sample shown in Fig. 2A. (A-C) The benchmarking of our algorithm compared to random search, MAP-elites[20] (ME), ME operating on an offline trained surrogate model (ME-SM) (inspired from the works[44-49]), and deep surrogate assisted map-elites[25] (DSA-ME). The runs are done for five seeds for random and ten seeds for the other implemented algorithms from the literature, the solid lines represent the mean, and shaded areas represent the 1.96-sigma range of standard deviation **(A)**. The performance range is clipped for the range of [2, 4.5] to have a close up view on the algorithms of this work and ME **(B)**. The performance range and the number of evaluations is further clipped for the range of [3, 4.5] and [1e5, 2.5e5],

respectively, where the NN-guided operation starts for our algorithm. This magnified view highlights the greater performance improvement (~37%) and faster rate (~33%) of our algorithm compared to pure MAP-elites **(C). (D-I)** Comparison of each algorithm in terms of the performance metrics of final performance, quality diversity score (QD-score), global performance, reliability, precision, and coverage (SI S1.3). Performance accounts for the final best design **(D)**; QD-score measures the quality and diversity of the archive by calculating the sum of performance values for all the cells **(E)**; global performance compares the best-performing design by dividing the single best-performing design of a run with the best-possible design found by any algorithm in any run **(F)**; reliability assesses the average quality of the generated designs for each cell by dividing them with the best-performing design found by any algorithm in any runs for the respective cell **(G)**; precision is similar to reliability but differs by ignoring the cells that are not filled by the algorithm **(H)**; coverage is a measure of how well the overall design space is explored for the defined parameters of interest map **(I)**. The gray dot and error bars represent the mean and one-sigma range of standard deviation, respectively.

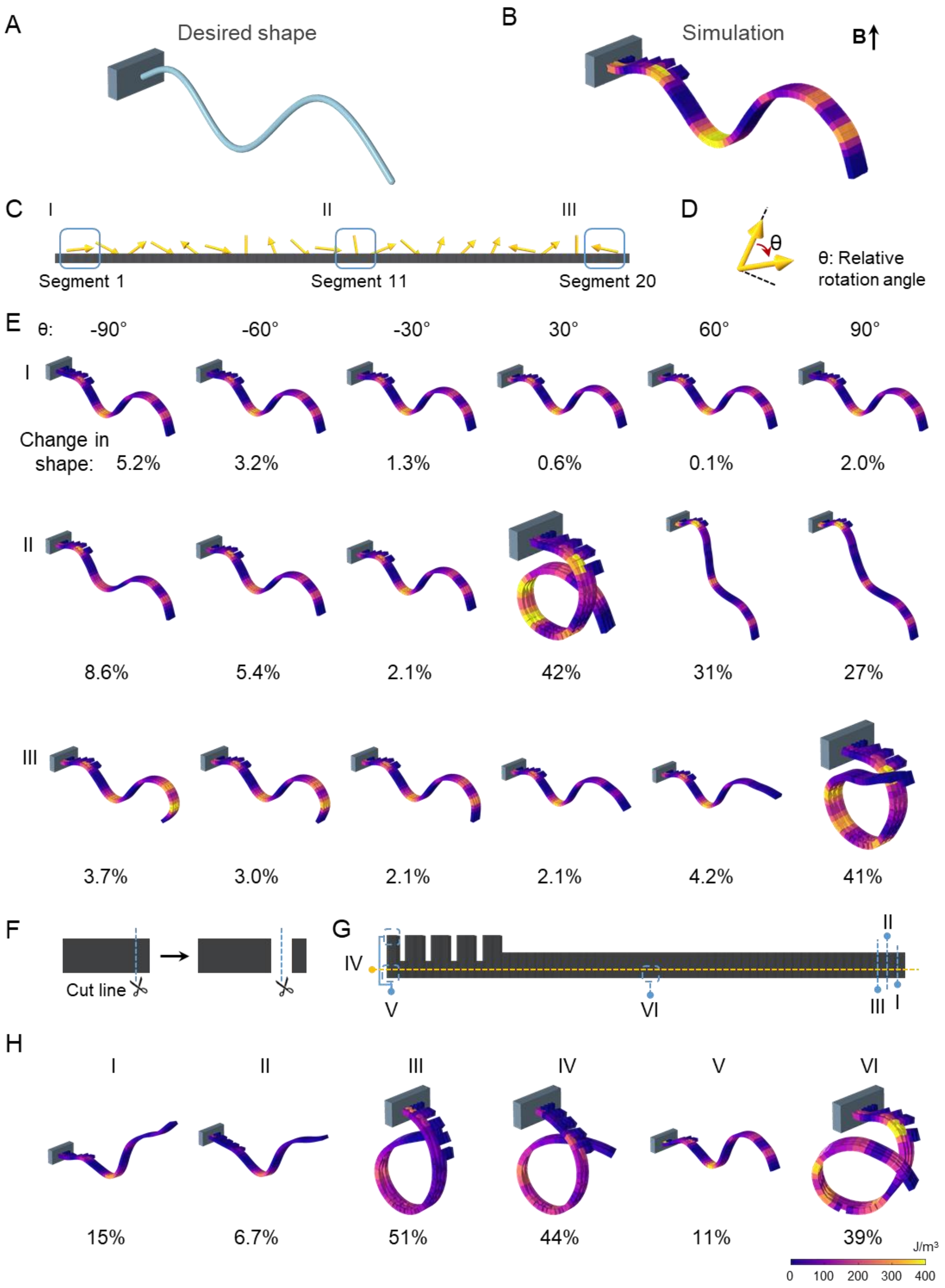

Figure S6. Sensitivity analysis for the effect of magnetization and morphology on shape-morphing of magnetic soft material. (A, B) Conceptual drawing of a soft beam structure with desired shape-morphing and simulation of the best-performing data-driven design. **(C, D)** Magnetic profile of the best-performing design from the side view for the desired

shape-morphing. Blue squares show the segments to be changed individually. Relative rotation angle (**θ**) is defined as the rotational change in the clockwise direction from the original magnetization orientation. The Relative rotation angle of the highlighted segments is separately changed from -90° to 90° in 30° increments to investigate the sensitivity of the magnetic profile for shape-morphing. **(E)** Simulation results, and the change in shape ratio for altered magnetic profiles after the rotated magnetizations of segment 1 (i), segment 11 (ii), and segment 20 (iii), emphasizing non-linear behavior and non-intuitive nature of the magnetic soft materials. **(F)** Effect of the morphology on shape-morphing is studied by cutting selected voxels of the original design morphology. Cuts are shown by the dashed lines. **(G)** Selected separate cut cases determined as a cut of one voxel line (I), two voxel lines (II), three voxel lines (III) from the beam tip, one lateral voxel line (IV), voxels from the sides at the fixed side (V), and voxel at the middle part resulting in a cut out of 3, 6, 9, 60, 2, and 1 voxel, respectively. **(H)** Simulation results, and the change in shape-morphing ratio for the new morphologies after the cuts shown in (I - VI). Shape-morphing change ratio is calculated by the average displacement, with respect to the original design, normalized to the body length. Actuation is performed by applying a magnetic field (**B**) of 30 mT in the direction indicated by the black arrows. Magnetization directions are depicted via yellow arrows.

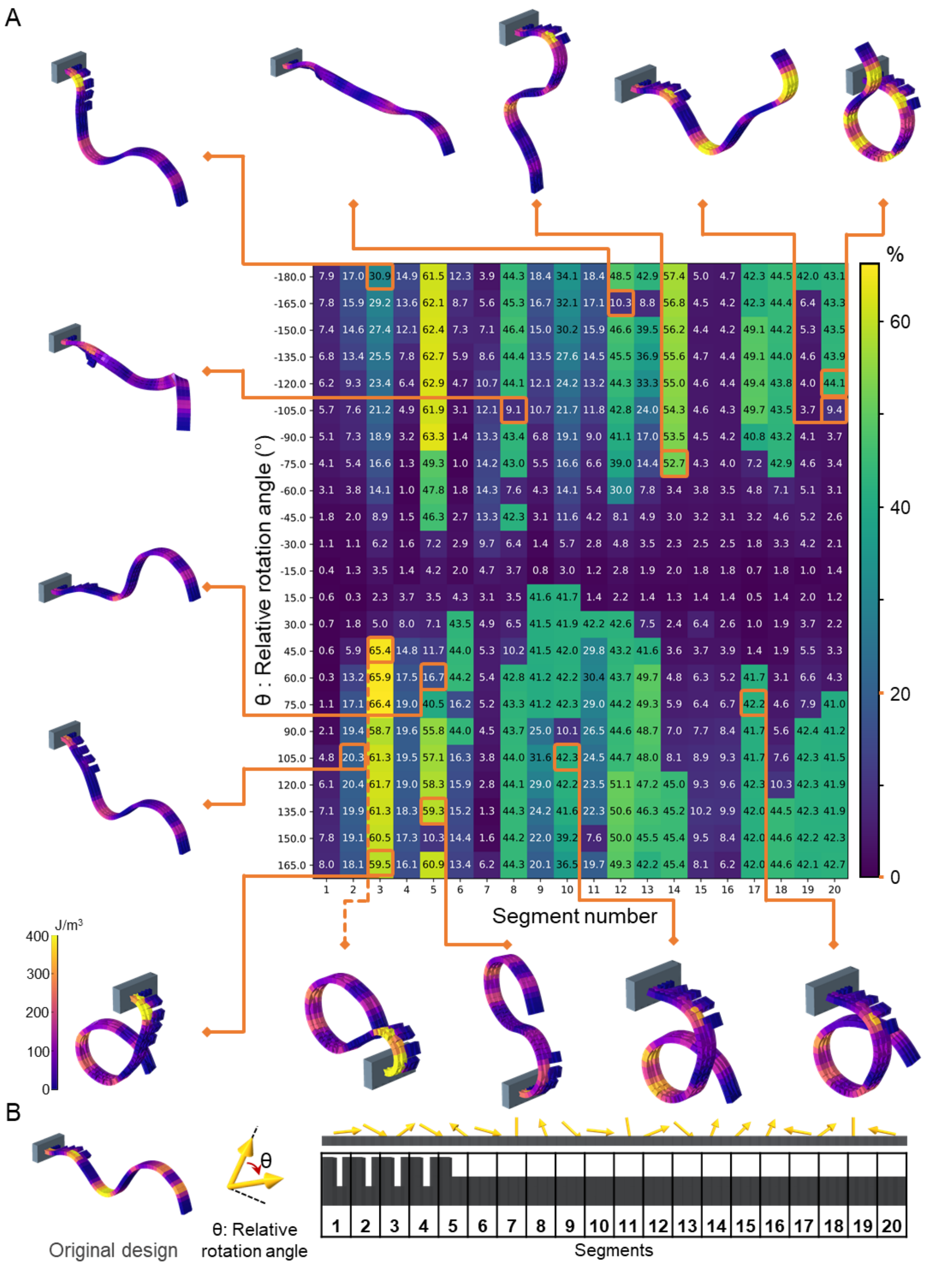

Figure S7. Sensitivity heatmap and selected examples for the effect of magnetization on shape-morphing of magnetic soft material. (A) The sensitivity heatmap showing the shape-morphing change ratio (%), for the demonstration in Fig. 2A, based on the relative

change of the magnetization direction (**θ**, y-axis) at different segment numbers (x-axis). The heatmap and illustrative cases highlight the non-linear and non-intuitive behavior of magnetic soft materials. The selected examples are marked with orange boxes, and their corresponding shape-morphing results are linked via orange lines. Shape-morphing change ratio is calculated as the average displacement error, with respect to the original design, normalized to the body length. Color bars represent the shape-morphing change ratio (%) and average strain energy density (J/m^3). **(B)** The shape-morphing of the original design is shown for comparison, along with the description of the relative rotation change (**θ**), and the respective segment numbers of the given original design.

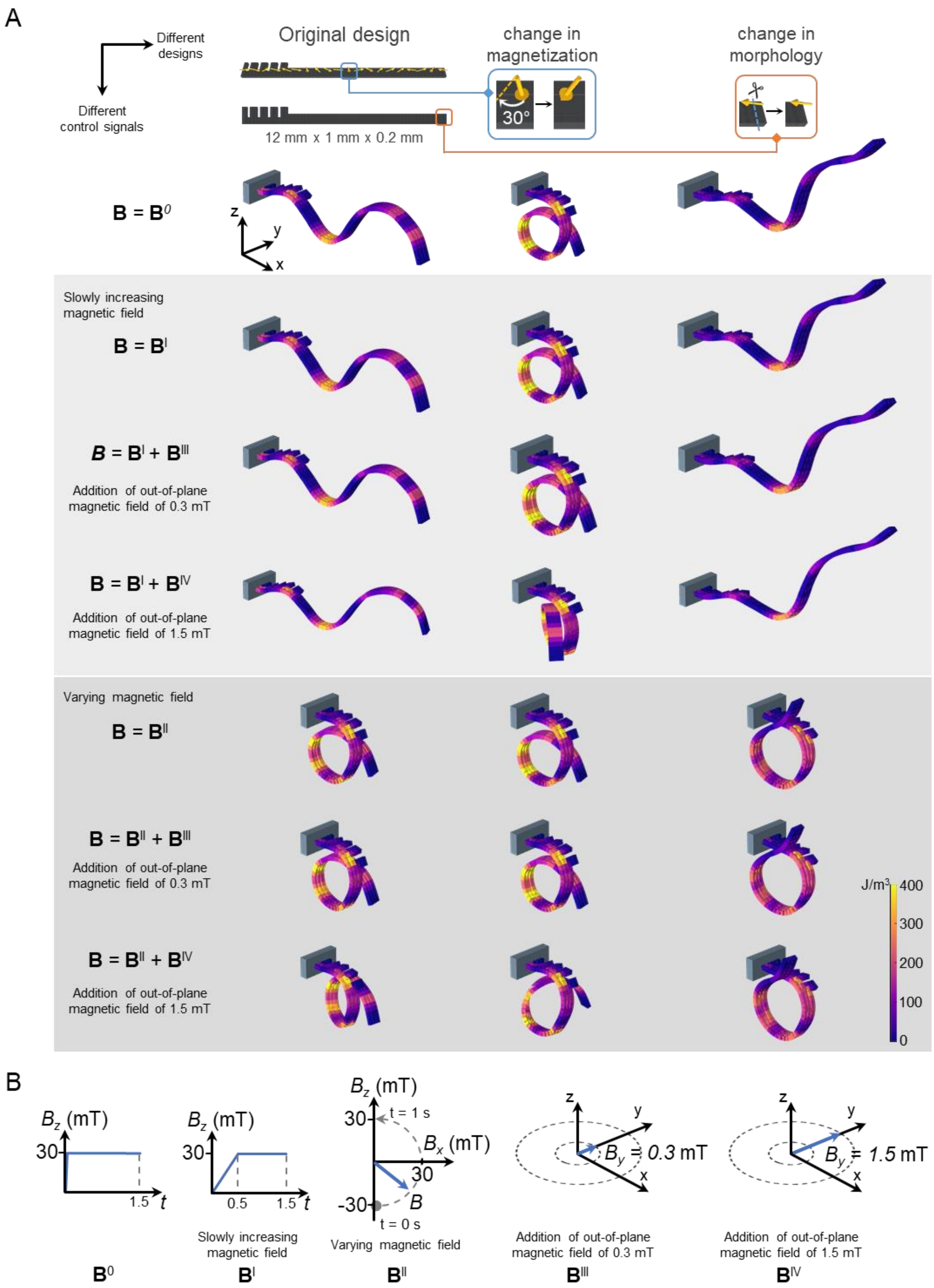

Figure S8. Sensitivity analysis for the effect of applied magnetic field on shape-morphing of magnetic soft beams. (A) Simulation results are presented for the original design (1st column), a disturbed magnetization profile (2nd column), and a disturbed

morphology (3rd column) under a range of external magnetic fields (rows). In the 2nd column, the magnetization of the 11th segment is changed 30°, corresponding to a 0.4% change in the overall magnetic profile, and in the 3rd column, the morphology of the design is changed by cutting a single voxel line laterally corresponding to a 1.5% change in overall morphology (Fig. 1B). **(B)** The rows represent different magnetic field configurations: the 1st row shows the original magnetic field with a sudden applied 30 mT ($\mathbf{B}^{0}$), the 2nd row shows a gradually increasing magnetic field ($\mathbf{B}^{I}$), the 3rd row includes a gradually increased magnetic field with a 0.3 mT out-of-plane in y direction component ($\mathbf{B}^{I}+\mathbf{B}^{III}$), 4th row includes gradually increased magnetic field combined with a 1.5 mT out-of-plane in y direction component ($\mathbf{B}^{I}+\mathbf{B}^{IV}$), 5th row shows a rotating magnetic field from -z to +z direction ($\mathbf{B}^{II}$), 6th row combines the rotating magnetic field from -z to +z direction with a 0.3 mT out-of-plane in y direction component ($\mathbf{B}^{II}+\mathbf{B}^{III}$), 7th row combines a rotating magnetic field from -z to +z direction with a 1.5 mT out-of-plane in y direction component ($\mathbf{B}^{II}+\mathbf{B}^{IV}$). Color bars indicate the average strain energy density.

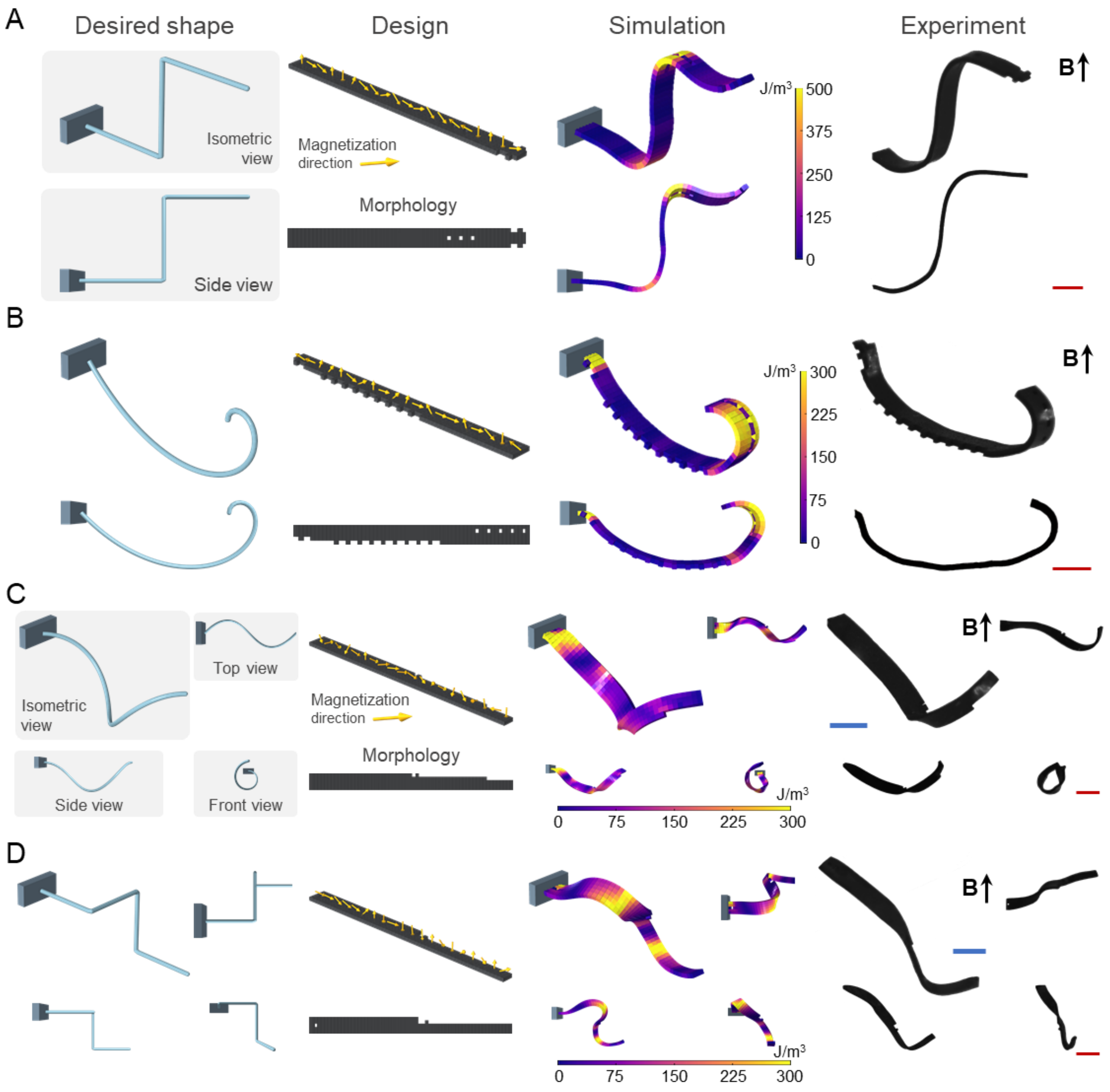

Figure S9. Data-driven design of morphology and magnetic profile for 2D and 3D shape-morphing magnetic soft beams. (A-D) The desired shape-morphing from different views depicted as averaged lateral voxel positions on the longitudinal axis, data-driven best-performing designs with morphology and magnetic profile, predicted shape-morphing colored with average strain energy density, and experimental shape morphing under an external magnetic field of 2D step signal **(A)**, Fibonacci spiral **(B)**, and 3D helix **(C)**, step **(D)** shapes. Design spaces for 2D and 3D shape-morphing are calculated as ~2.7e141, and ~3.4e186, respectively. Scale bars, 2 mm. Actuation is performed by applying a magnetic field (**B**) of 30 mT in the direction indicated by the black arrow. Magnetization directions are depicted via yellow arrows.

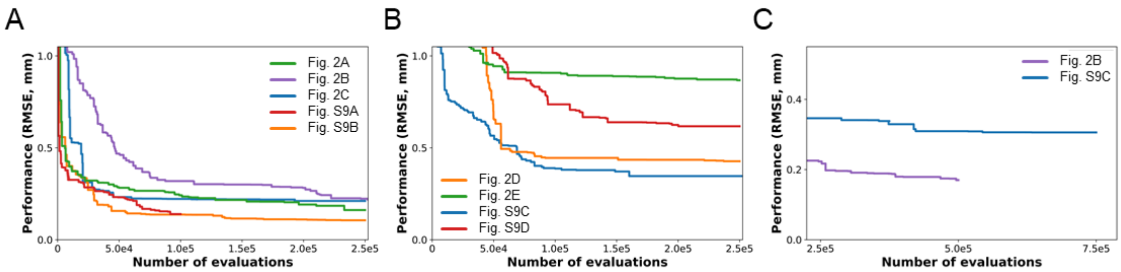

Figure S10. Evolution of performance for 2D and 3D shape-morphing magnetic soft beams during the data-driven design progress. (A, B) Evolution of the shape-morphing behavior for 2D **(A)** and 3D **(B)** magnetic soft beams with the performance in the range of [0, 1] and the evaluation range of [0, 2.5e5]. Botch cases show initial rapid improvements, followed by a slower but steady increase in performance. **(C)** Extended evolution of 2D shape-morphing (square) over 5e5 evaluations and 3D shape-morphing (helix) over 7.5e5 evaluations, further illustrating the steady performance gains.

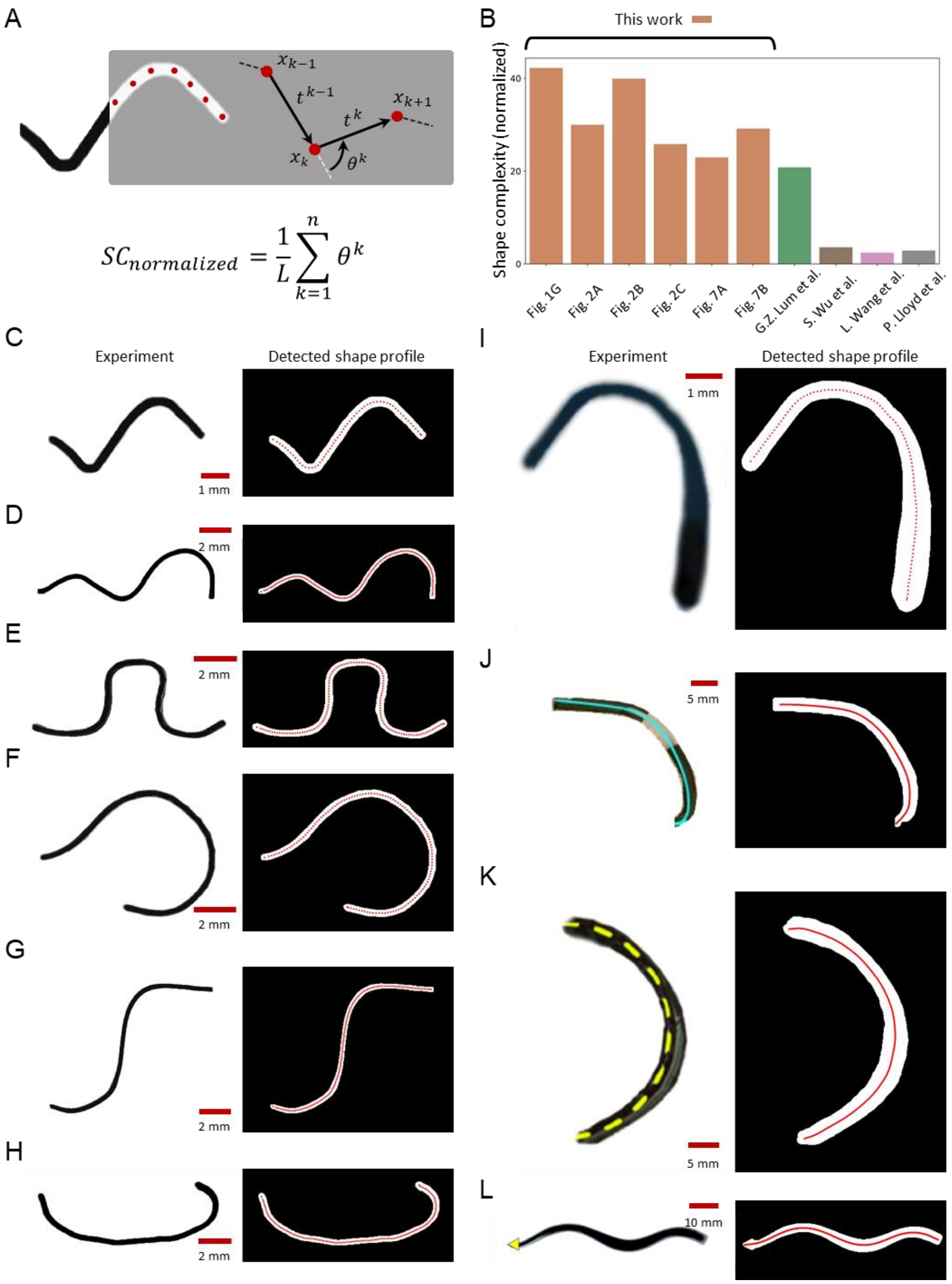

Figure S11. The shape complexity of 2D shape-morphing demonstrations in our work in comparison to the state-of-the-art in the literature. (A) Calculation of the normalized shape complexity score ($SC_{normalized}$) by automated detection of beam profile, discretization into 100 µm distanced nodes, summation of the angular change in vectoral directions among nodes, and dividing the obtained shape complexity score with the beam length for

normalization. **(B-L)** The comparison of the normalized shape complexity score **(B)** of the 2D shape-morphing demonstrations in Fig. 1G **(C)**, Fig. 2A **(D)**, Fig. 2B **(E)**, Fig. 2C **(F)**, Fig. S9A **(G)**, and Fig. S9B **(H)**, as well as, the state-of-the-art works of G.Z. Lum et al. [55] **(I)**, S. Wu et al. [47] **(J)**, L. Wang et al. [56] **(K)**, and P. Lloyd et al. [59] **(L)**.

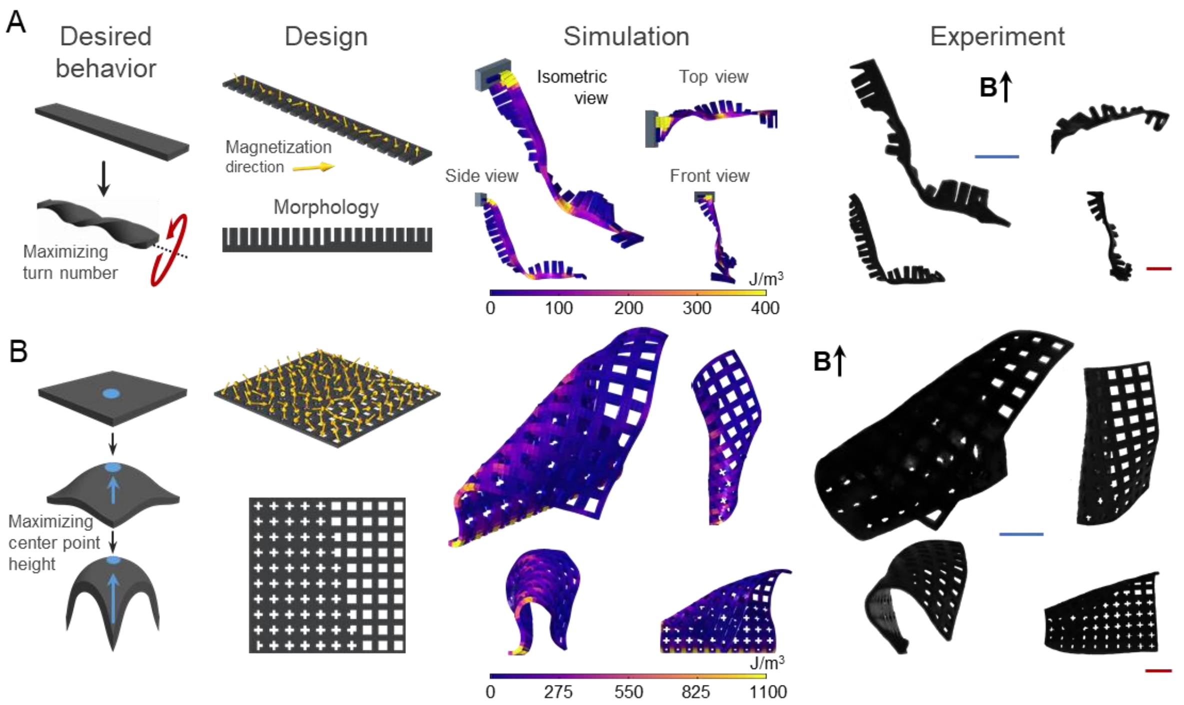

Figure S12. Data-driven design of morphological tasks in magnetic soft structures. (A-B) Conceptual drawing of the desired morphological tasks, data-driven best-performing designs with morphology and magnetic profile, predicted behavior, experimental realization under external magnetic field of maximizing the turn number along the longitudinal axis of a beam **(A)**, and maximizing the height of the center point of a sheet **(B).** Design spaces for the soft structures are ~3.4e186, and ~5.4e1233, respectively. Scale bars, 2 mm. Actuation is performed by applying a magnetic field (**B**) of 30 mT in the direction indicated by the black arrow. Magnetization directions are depicted via yellow arrows. Color bars indicate the average strain energy density.

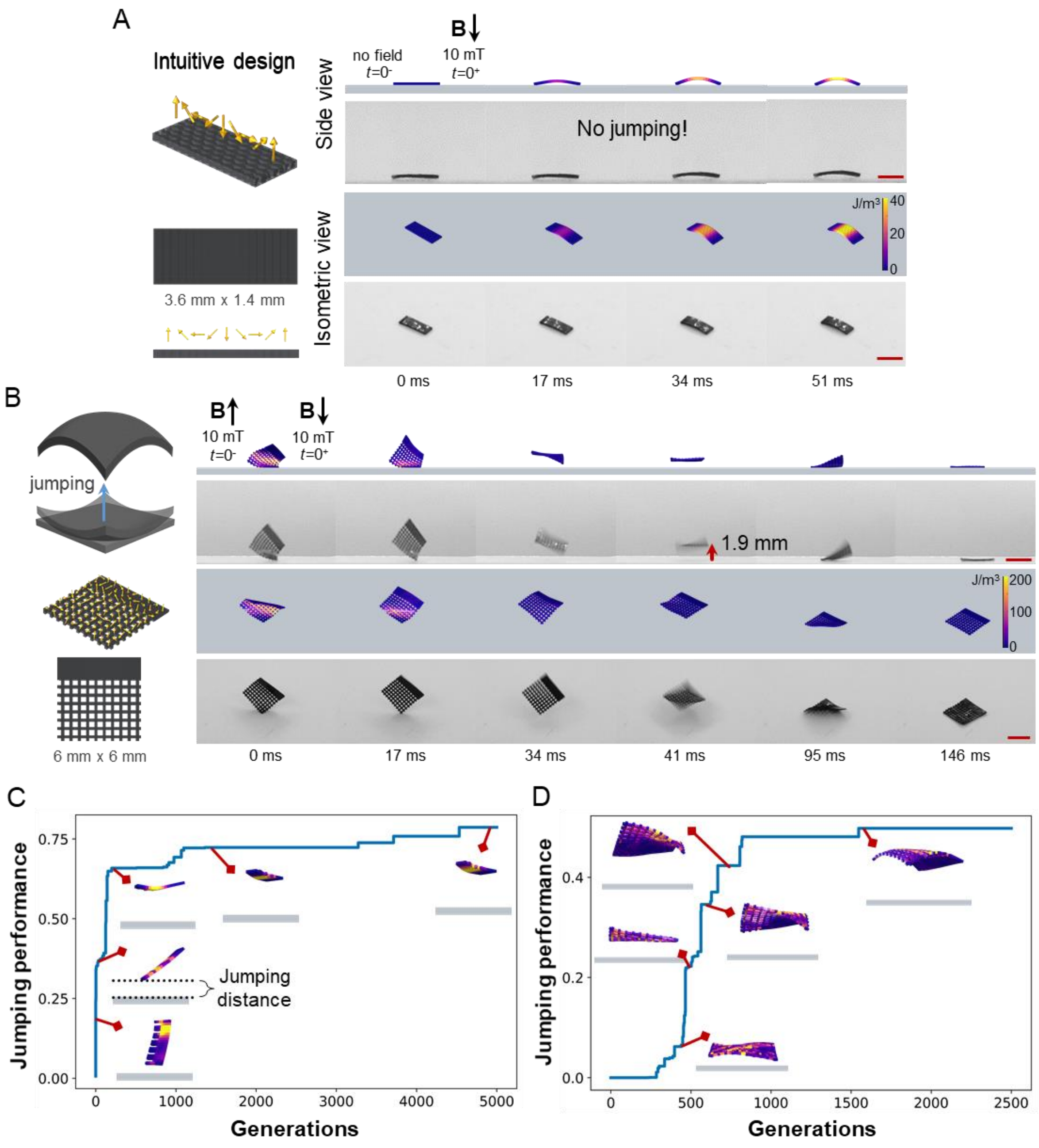

Figure S13. Data-driven design of jumping magnetic soft structures. (A) Intuitive magnetic soft robot design adapted from the literature[53] with a continuous magnetic profile and a uniform beam morphology. Dynamic behavior of the intuitive design in simulation and experiment, as shown in side (top row) and isometric (bottom row) views. The intuitive design failed to generate any jumping behavior as predicted by the computational simulation. **(B)** Conceptual drawing of jumping behavior for a magnetic soft robot with a square-shaped sheet. Data-driven magnetic soft robot design with a discrete magnetic profile incorporating arbitrary

changes of magnetization in adjacent segments and non-uniform morphology. Predicted and experimental dynamic behavior of the soft robot are shown in side (top row) and isometric (bottom row) views, achieving ~1.9 mm (0.43 body length) jumping height. Design space is ~1.2e752 for sheet robot. Scale bars, 2 mm in **(A)**, and 4 mm in **(B)**. Actuation is performed by applying a uniform magnetic field (**B**) of 10 mT in the direction indicated by the black arrows. Magnetization directions are depicted via yellow arrows. **(C, D)** Evolution of the jumping behavior performance for magnetic soft beam **(C)** and sheet **(D)**. Jumping performance is defined as the minimum distance of the magnetic soft robot to the surface at peak. Insets highlight the progression of simulated best-performing designs over the generations. Initial quick improvements, followed by a slower but steady increase in performance are observed for both beam and sheet jumping behaviors.

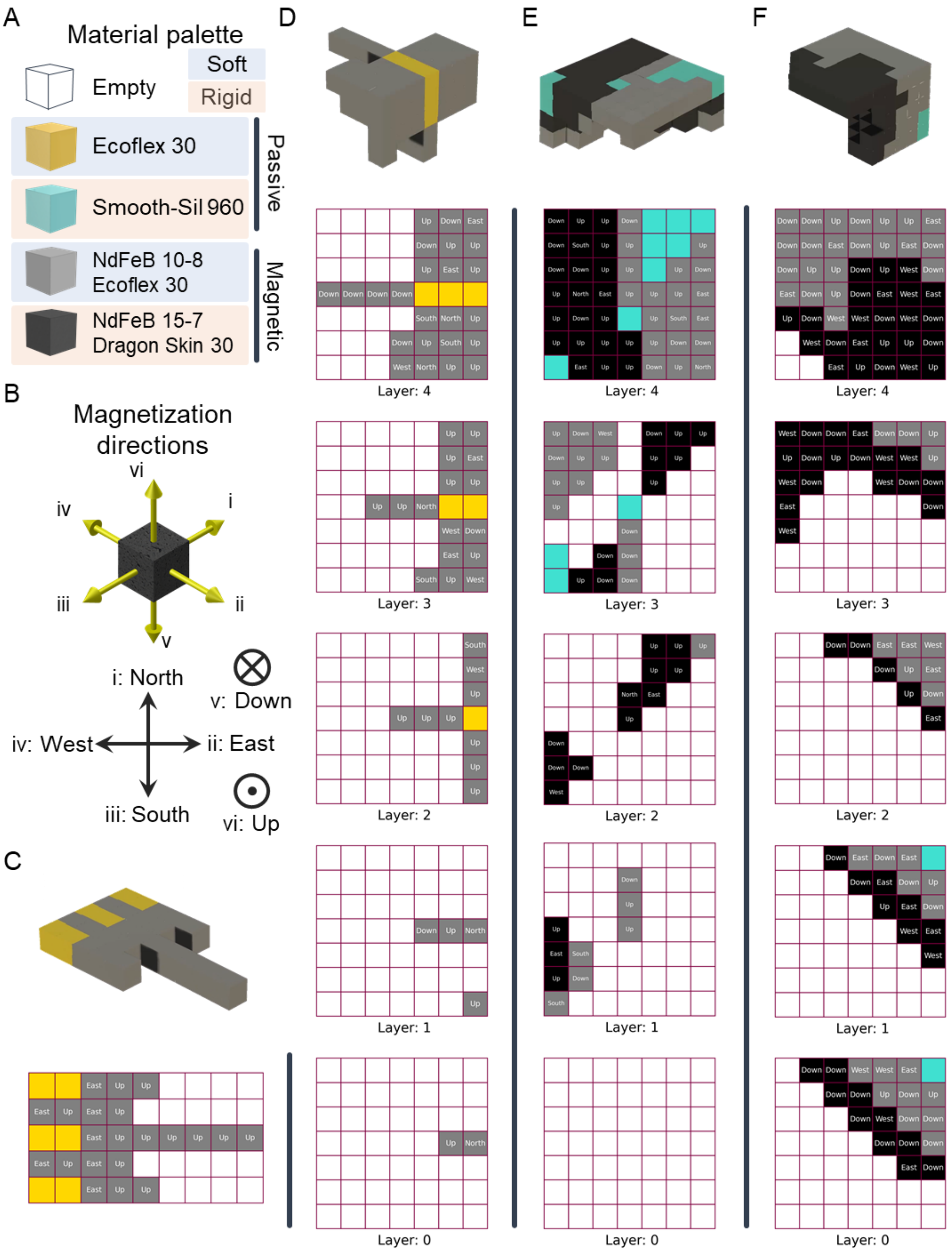

Figure S14. Design details of 2D and 3D multi-material soft robots. (A) The material palette consists of five types, including a cut out “empty” voxel, two passive and two magnetically responsive materials with soft and rigid variations, depicted via blue and orange backgrounds, respectively. **(B)** The programming directions of the magnetic materials,

depicted via yellow arrows, defined for each voxel by the six discrete primary cartesian axes directions, namely North (i), East (ii), South (iii), West (iv), Down (v) and Up (vi). **(C-F)** The overall structural material composition and programming directions of magnetic voxels shown layer by layer for vertical **(C, D)** and directional **(E)** jumping, as well as traversing **(F)** 2D (C) and 3D (D-F) multi-material soft robots. The layers, which are defined as slices taken from x-y plane, are arranged in columns starting with the top layer (layer 4) towards the bottom (layer 0). The voxel colors indicate the material type chosen from the material palette by our data-driven design algorithm. The programming directions of the magnetic materials stated for each voxel.

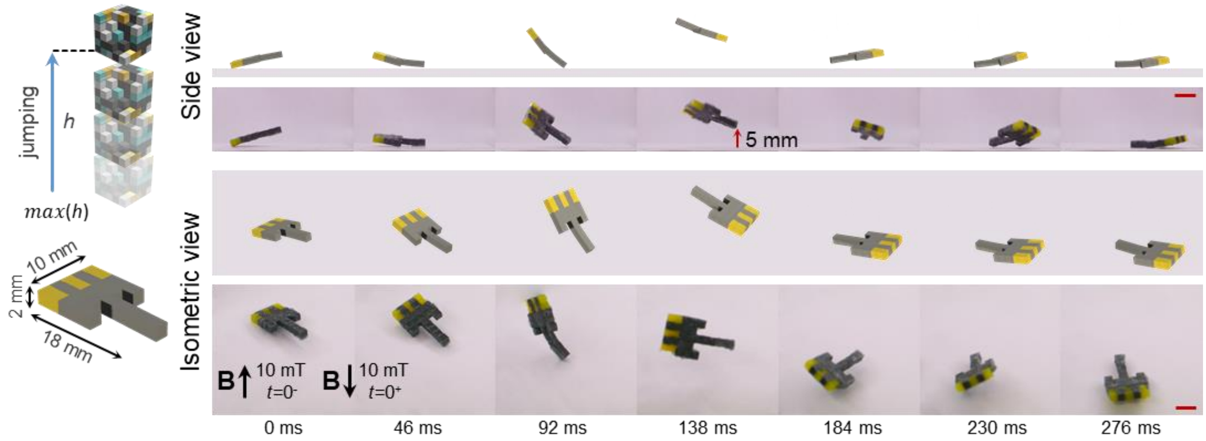

Figure S15. Multi-material 2D structural design of magnetic soft millirobots. The conceptual drawing of the desired robotic behavior, best-performing structural design, and predicted behavior and experimental realization shown in side (top rows) and isometric (bottom rows) views for vertical jumping multi-material soft robots, achieving ~5mm (0.28 body length) jumping height. Design space is calculated as ~2.9e66. Scale bars, 5 mm. Actuation is performed by applying a uniform magnetic field (**B**) of 10 mT in the direction indicated by black arrows. Voxel colors indicate the material types.

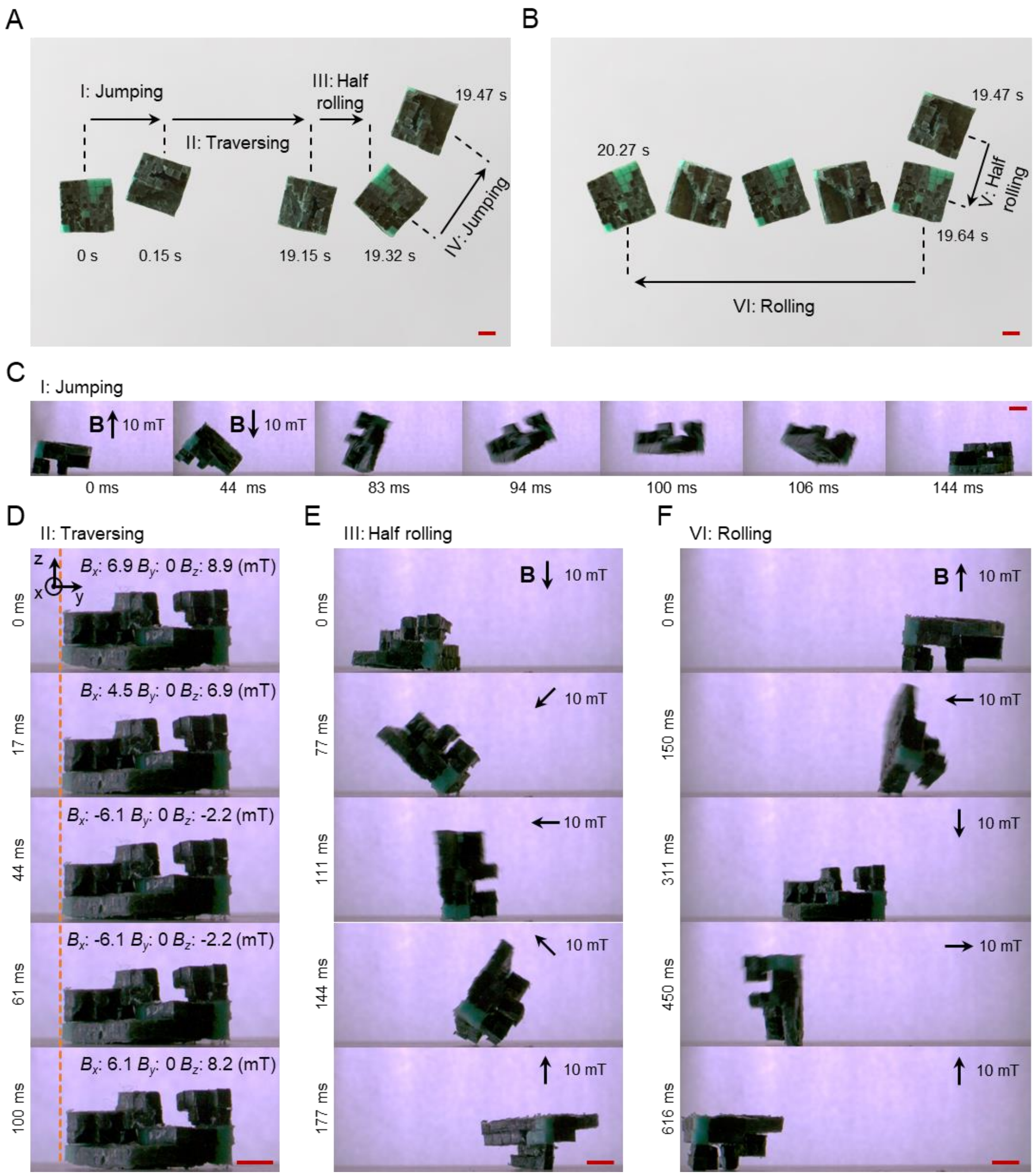

Figure S16. Multimodal locomotion of the 3D multimaterial robot (Fig. 4D) on a pre-defined path. (A-B) The overview of multimodal locomotion of our 3D multimaterial robot (Fig. 4D) on a pre-defined path performing jumping (I), traversing (II), half rolling (III), and jumping (IV) to reach the desired end position **(A)**, followed by half rolling (V), and rolling (VI) behaviors to get back to the starting position **(B)**. The demonstrations are shown from top view. **(C-F)** The selected multimodal behaviors of jumping **(C)**, traversing **(D)**, half rolling **(E)** and backward rolling **(F)** achieved by the changes in the control signal (magnetic fields). The orange dashed

line is provided to highlight the change in the position of the robot in **(D)**. Scale bars, 4 mm. Actuation is performed by applying a uniform magnetic field (**B**) in the direction indicated by black arrows with 10 mT strength (C, E, F), or in the explicitly mentioned B vector (Bx, B_y, B_z) direction and strength (D).

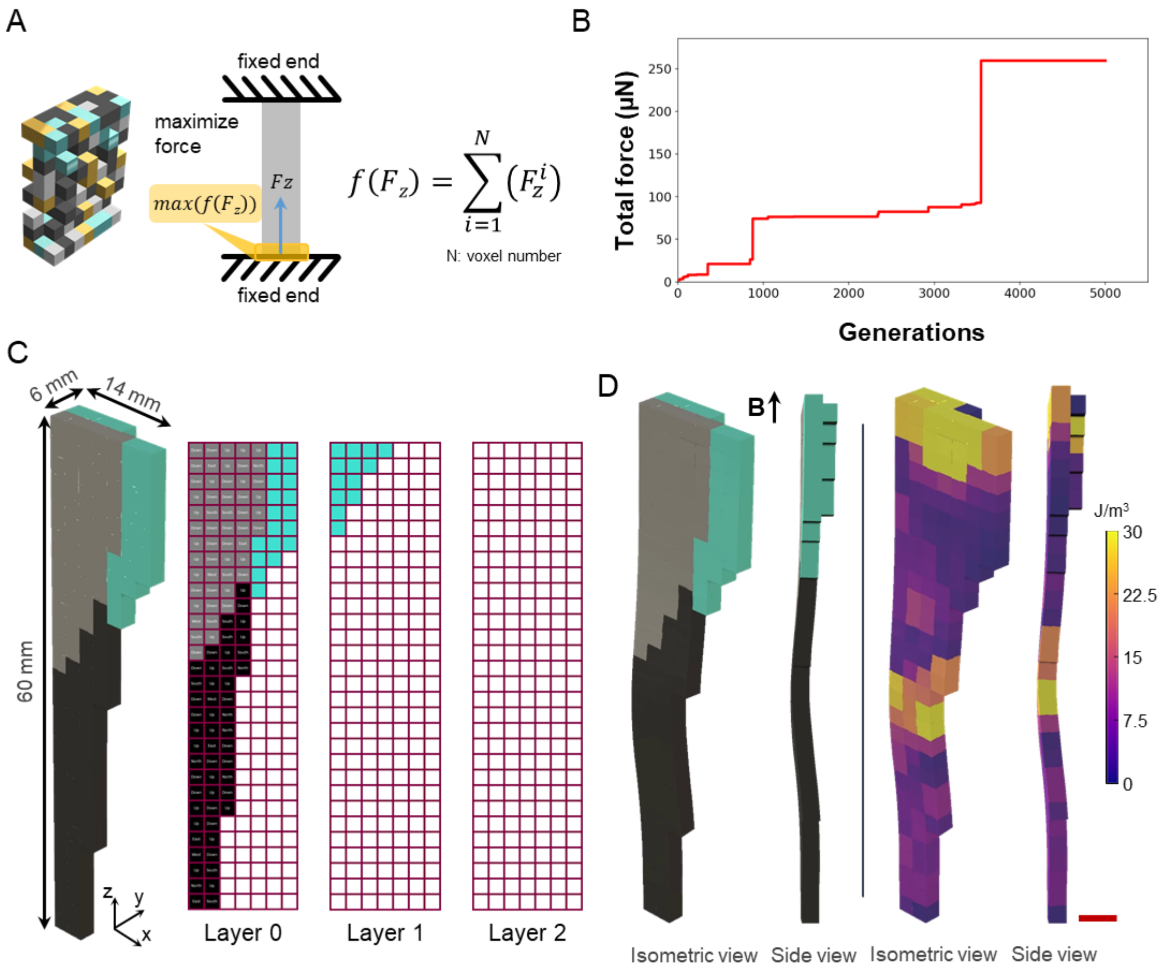

Figure S17. Multi-material and 3D structural design of magnetic soft structure for maximizing force generation. (A) The conceptual drawing of the desired task for maximizing the force generation in z direction. The objective function is defined as total force in z direction (F_z) for the voxels at the bottom fixed end (highlighted with the yellow frame). **(B)** The best performing objective function results vs. generations is depicted, showcasing a total force of ~260 μN in z direction for the best design achieved after 5000 iterations, 2.5e5 evaluations. **(C)** The overall structural material composition and programming directions of magnetic voxels shown layer by layer for the best-performing design. Layers are taken from x-z plane, and voxel colors indicate the material types, as explained in Fig. 4 and Fig. S14. **(D)** The predicted behavior is shown in isometric and side view with voxels indicating material types (left column) and average strain energy density (right column). Design space is calculated as ~3.9e930. Scale bar, 4 mm. Actuation is performed by applying a uniform magnetic field (**B**) of 10 mT in

the direction indicated by the black arrow. Color bar indicates the average strain energy density.

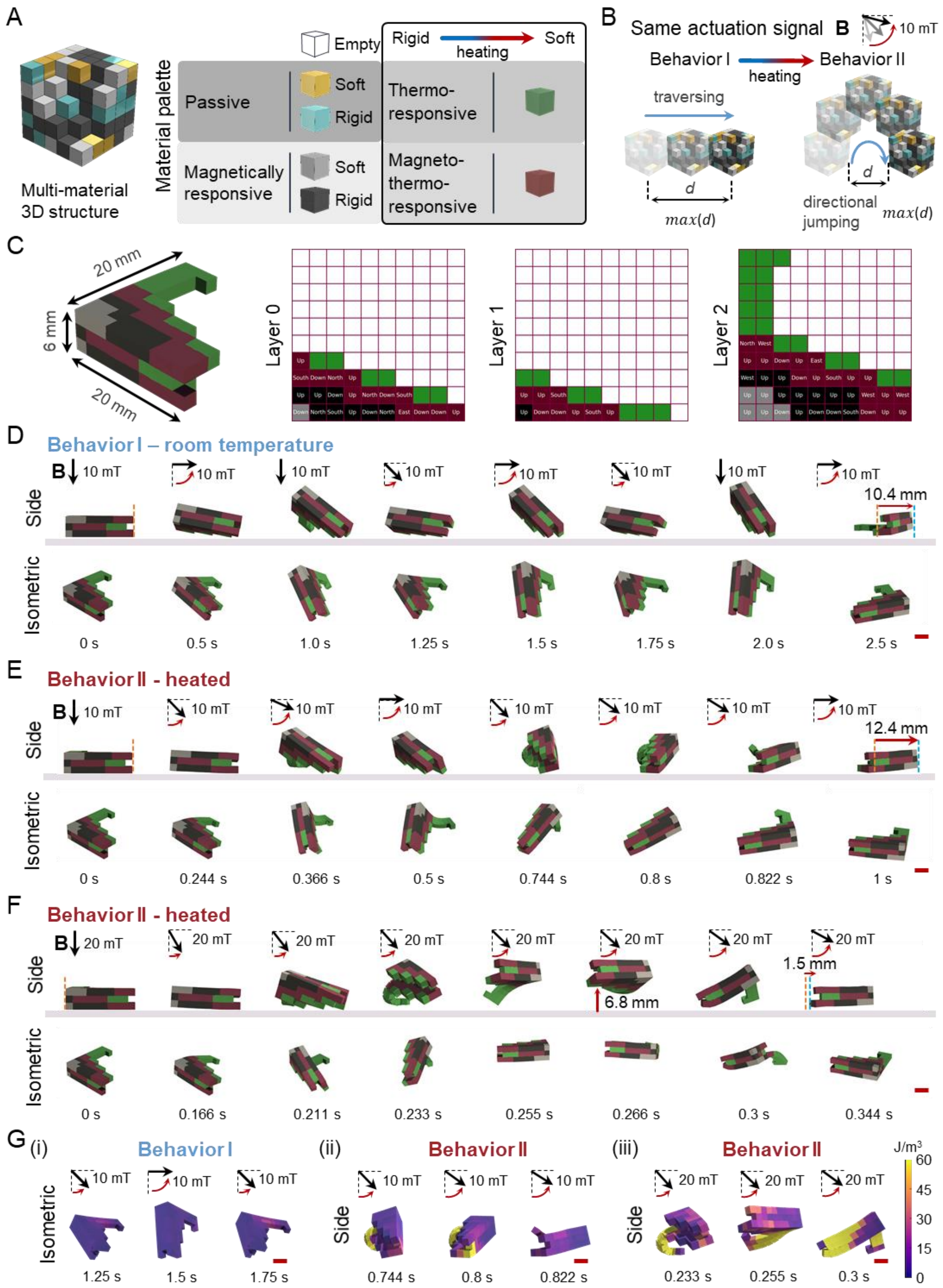

Figure S18. Design of configurable magneto- and thermo-responsive soft millirobots with 3D multi-material composition. (A) 3D and multi-material structural design with a material palette consisting of seven types. Thermo-responsive and magneto-thermo

responsive materials, that are rigid in room temperatures and become soft after heated, are added to the palette in addition to the cut out “empty” voxel, two passive and two magnetically responsive materials with soft and rigid variations. The magnetization directions of the magnetically responsive materials are restricted to six discrete primary cartesian axes directions. **(B)** The conceptual drawing of the desired robotic configurable behavior of traversing and directional jumping under the same magnetic actuation signal with thermally triggered behavior change. **(C)** The best-performing structural design shown in isometric view together with detailed structural material composition and programming directions of magnetic voxels shown layer by layer. Voxel colors indicate the material types. **(D, E)** The predicted behavior of a magneto-thermo-responsive soft robot in side (top rows) and isometric (bottom rows) views for traversing (behavior I) at room temperature **(D)**, directional jumping (behavior II) after heating **(E)** under the same magnetic actuation signal, achieving ~2.1 mm (0.1 body length) traversing per cycle, and ~12.4 mm (0.62 body length) directional jumping distance, respectively. **(F)** Best performing design for configurable behavior demonstrated vertical jumping after heating when the applied magnetic field strength is doubled (~6.8 mm, 0.34 body length jumping height), indicating the uniqueness of designs generated by our data-driven strategy for desired behaviors under pre-defined control inputs. Voxel colors indicate the material types. Orange and blue dashed lines represent initial and final reference position, respectively. **(G)** Decreased elastic modulus triggered by heating results in drastically increased average strain energy density over the body of the same soft robot for **(ii, iii)** jumping behaviors compared to **(i)** traversing behavior. Color bar indicates the average strain energy density. Design space is calculated as ~9.4e486. Scale bars, 4 mm. Actuation is performed by applying a uniform magnetic field (**B**) in the direction indicated by black arrows.

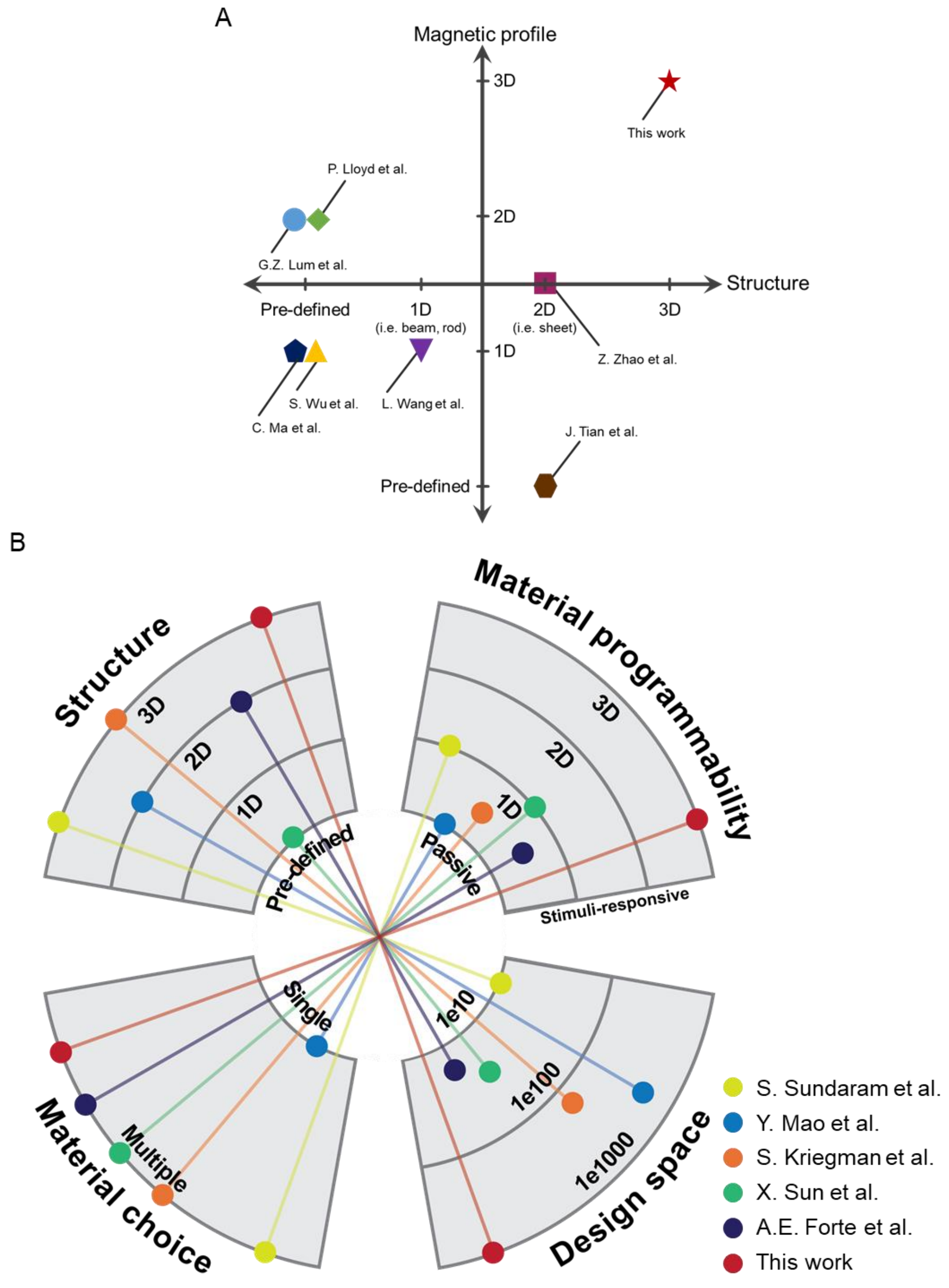

Figure S19. Performance comparison of previously reported design strategies with the current study. (A) Design optimization strategies of this work and the works of G.Z. Lum et

al.[55], S. Wu et al.[47], L. Wang et al.[56], P. Lloyd et al.[59], J. Tian et al.[58], C. Ma et al.[46] and Z. Zhao et al.[60] are compared exclusively for magnetic soft materials. The magnetic profile indicates the degree of freedom available in magnetization directions for the optimization. The structure describes the design domain of the optimization in spatial space**. (B)** The capabilities of our data-driven design approach are compared with the prominent existing methods for soft and stimuli-responsive soft materials introduced by S. Sundaram et al.[76], Y. Mao et al.[70], S. Kriegman et al.[5], X. Sun et al.[44], and A.E. Forte et al.[49]. The structure is the available spatial space for design. Material programmability refers to the degrees of freedom available for the related programmed stimuli-responsive material(s). Material choice demonstrates the available material types for the design. Design space is approximated with the reported parameter numbers and the discretization of continuous parameters, highlighting the complexity of the design problem in related works.

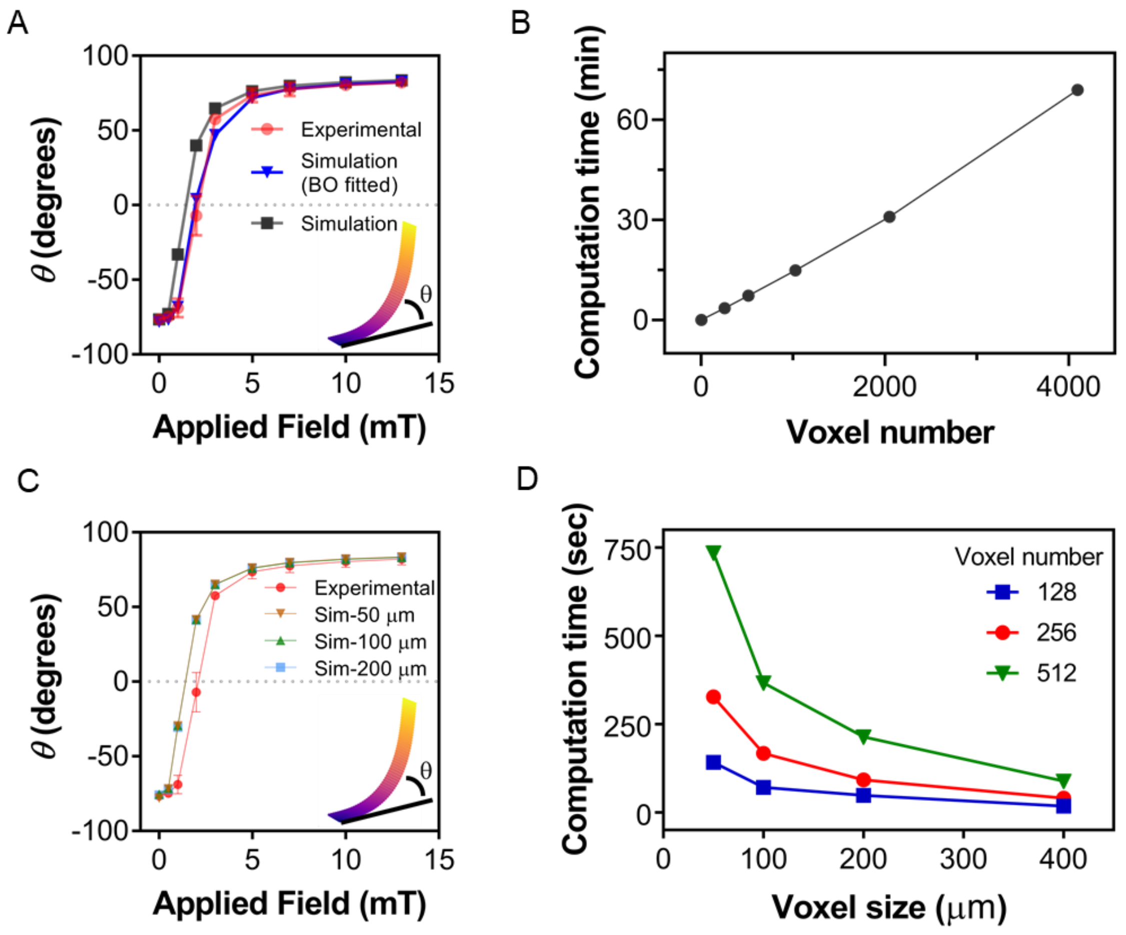

Figure S20. Validation and characterization of the computational predictive model. (A) Validation experiments are repeated for seven beam samples (12 mm length x 1.6 mm width x 0.2 mm thickness). Simulation results are obtained with the measured experimental parameters of magnetic material density, elastic modulus and magnetization strength. The accuracy of the simulation is further improved by fitting parameters of the elastic modulus and magnetization strength to the validation experiments utilizing Bayesian optimization (BO) method. Deflection angle (**θ**) values are obtained with respect to the beam tip position. Beam is magnetized along the longitudinal axis, fixed from one end at the horizontal position, and uniform magnetic field is applied vertically via a coil setup in Helmholtz configuration. **(B)** Simulation computation time increases with respect to the total voxel number, scaling linearly. Voxel size is fixed to 200 µm, and 1-second-long dynamic simulations are evaluated with voxel numbers of 4096, 2048, 1024, 512, 256, and 128. **(C)** The effect of voxel resolution on the validation accuracy is investigated, for voxel sizes of 50 µm, 100 µm, and 200 µm. No

significant change is observed in the validation accuracy, showing voxel size of 200 µm can be used without loss of generality. **(D)** Simulation computation time with respect to voxel size is characterized by a total number of 128, 256, and 512 voxels. A decrease in voxel size (or increase in resolution) increases the computational time exponentially.

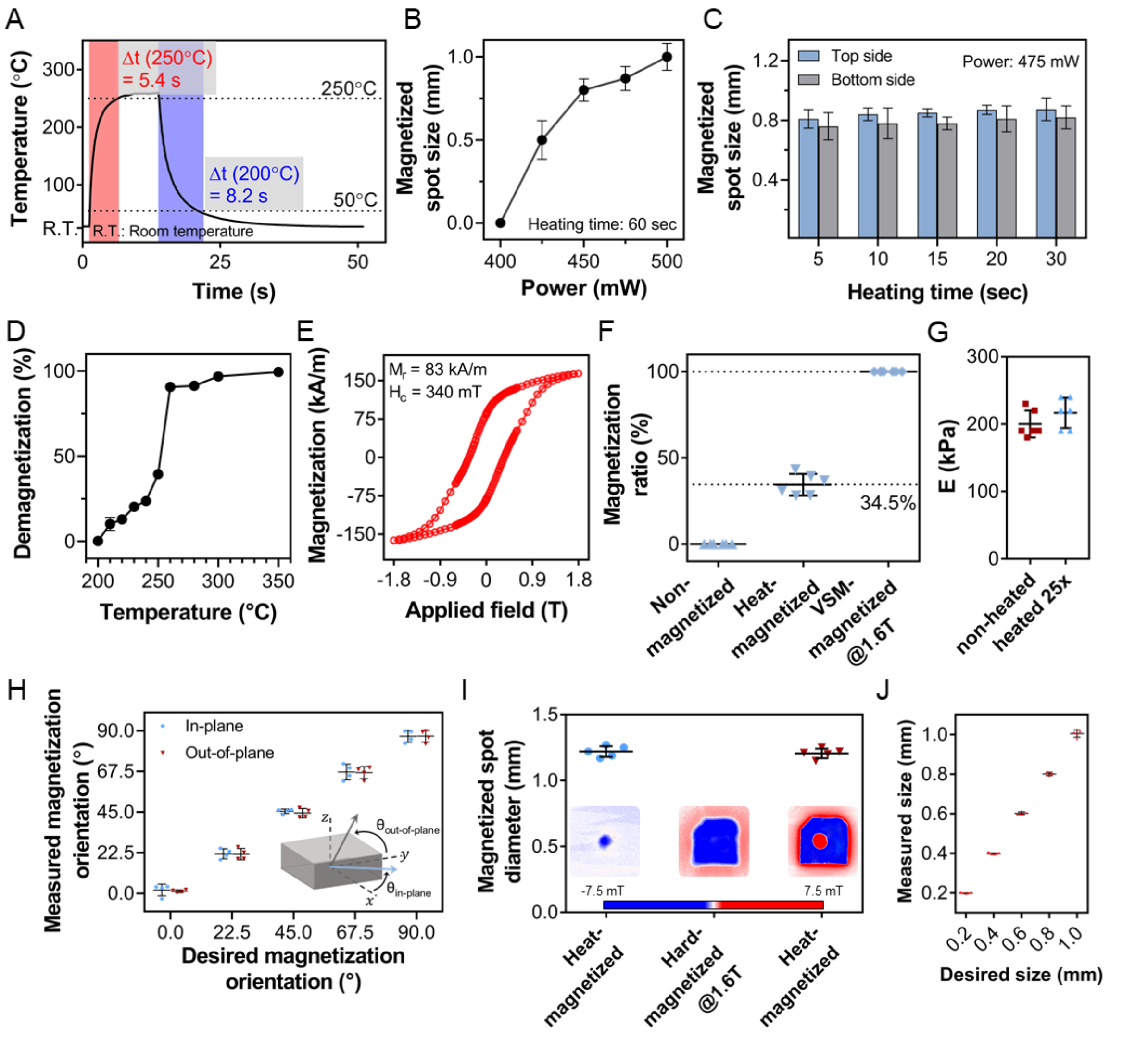

Figure S21. Magnetic, mechanical and photothermal properties of magnetic soft material. (A) Heating and cooling cycle under the effect of NIR laser heating. **(B)** Effect of laser power on the magnetized spot size. **(C)** Effect of heating time on the magnetized spot size both on the bottom and laser applied top surfaces. **(D)** Effect of heating temperature on the demagnetization ratio, indicating the Curie temperature. **(E)** Hysteresis loop of magnetic soft material (mixture of $Nd_2Fe_{14}B$, MPFP 10-8, Ecoflex-30) at room temperature. Remanent magnetization (M_r) is 83 kA/m and coercivity (H_c) is 340 mT. **(F)** Magnetization ratio of heat-assisted magnetization after magnetizing at 250℃. Magnetization values for samples magnetized under a uniform 1.6 T field are considered as 100%. **(G)** Elastic modulus (E) of magnetic soft elastomers in native condition and after a heating cycle of 25 at 250℃. **(H)** The magnetization orientation accuracy of heat-assisted magnetization method for in-plane and

out-of-plane orientations characterized in the range of 0° to 90° in 22.5° increments. **(I)** The investigation on the effect of the surrounding magnetization on the heat-assisted magnetized spot size. The non-magnetized samples are magnetized via laser heating, hard-magnetized at 1.6 T, and magnetized in reverse direction via laser heating. The spot sizes of the laser-magnetized regions are characterized by the out-of-plane magnetic flux profile measurements via magneto-optical sensor (MagViewS, Matesy, Jena, Germany). Color bars indicate the magnetic flux density strength. **(J)** Laser micromachining precision of magnetic soft materials for square-shaped cuts with 0.2 mm, 0.4 mm, 0.6 mm, 0.8 mm, and 1mm edge lengths. Error bars depict the one-sigma range of standard deviation.

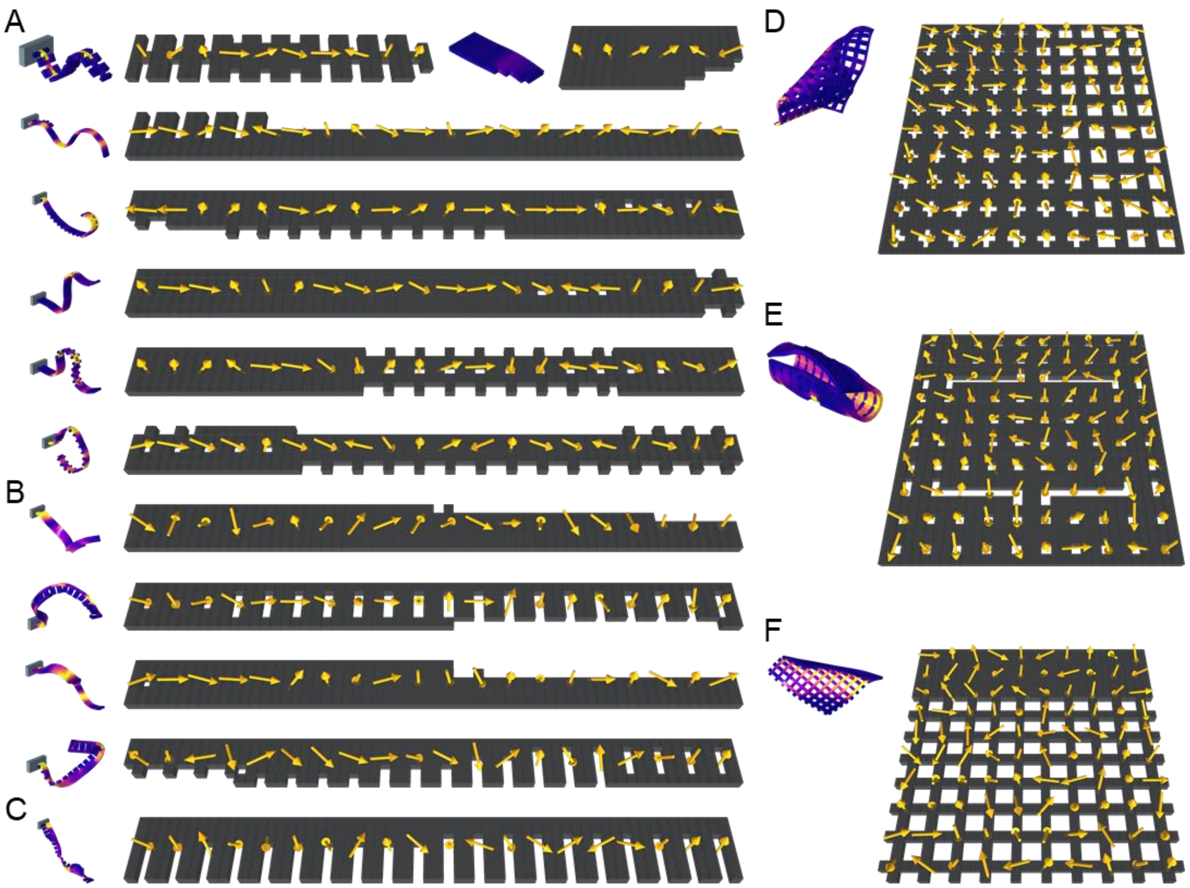

Figure S22. Data-driven design details of magnetically-responsive demonstrations. (A - F) Simulation results, magnetic profile and morphology of the beams with 2D magnetic profile **(A)**, beams with 3D magnetic profile **(B, C)**, and sheets with 3D magnetic profile **(D - F)**.

Supplementary tables

Demos	Shape complexity score (degrees)	Beam length (mm)	Normalized shape complexity score (degrees/mm)
**Fig. 1G**	253.5	6	42.2
**Fig. 2A**	359.9	12	30.0
**Fig. 2B**	479.3	12	39.9
**Fig. 2C**	309.9	12	25.8
**Fig. S9A**	275.5	12	23.0
**Fig. S9B**	350.1	12	29.2
**G.Z. Lum et al.** [55]	208.2	10	20.8
**S. Wu et al.** [47]	179.2	50	3.6
**L. Wang et al.** [56]	145.7	60	2.4
**P. Lloyd et al.** [59]	173.2	60	2.9

Table S1. Shape complexity comparison of 2D shape-morphing demonstrations with the state-of-the-art in literature. The shape complexity scores, beam length and the normalized shape complexity scores are provided for all of the 2D shape-morphing demonstrations in this work (Fig. 1G, Fig. 2A, Fig. 2B, Fig. 2C, Fig. S9A, and Fig. S9B), as well as, the state-of-the-art works of G.Z. Lum et al. [55], S. Wu et al. [47], L. Wang et al. [56], and P. Lloyd et al. [59].

Demo	Desired shape vs. Simulation		Simulation vs. Experiment	
	Position RMSE (µm)	Body-length ratio (%)	Position RMSE (µm)	Body-length ratio (%)
**Fig. 1F-G**	93.2	1.55	97	1.62
**Fig. 2A**	161	1.34	228	1.90
**Fig. 2B**	142	1.42	74	0.62
**Fig. 2C**	211	1.76	729	6.08
**Fig. S9A**	138	1.16	667	5.56
**Fig. S9B**	106	0.88	255	2.13
**Fig. 2D**	427	3.56	-	-
**Fig. 2E**	867	7.23	-	-
**Fig. S9C**	306	2.55	-	-
**Fig. S9D**	617	5.15	-	-

Table S2. Quantitative comparison of shape-morphing demonstrations for simulation and experimental results. The position errors for 2D and 3D shape-morphing demonstrations in terms of position (µm) root-mean-square error (RMSE) and body-length ratio. The percent body-length ratio is calculated by dividing the RMSE value to the beam length, and multiplied by 100. The 1st column (desired shape vs. simulation) provides information regarding the discrepancy between the desired shape and the simulation results and the 2nd column (simulation vs. experiment) quantifies the sim2real transfer success. The quantitative results for the comparison of simulation and experiment results are only reported for 2D shape-morphing demonstrations due to the fact that twisting and bending in 3D makes the accurate position extractions difficult from experimental images.

		Design with pre-defined morphology		Design of magnetic profile and morphology	
	Magnetic profile discretization	5 degrees	1 degree	5 degrees	1 degree
Demonstrations	Fig. 1F-G	3.7e18	3.7e25	5.3e63	5.2e70
	Fig. 2A-C<br>Fig. S9A-B	1.4e37	1.3e51	2.9e127	2.7e141
	Fig. 2D-E<br>Fig. S9C-D<br>Fig. S12A	1.9e68	1.7e96	3.8e158	3.4e186
	Fig. 3A<br>Fig. S13B	2.3e341	1e481	1e612	1.2e752
	Fig. 3D	1.4e11	2.2e15	1.2e49	1.8e53
	Fig. S12B	2.3e341	1e481	8.7e1093	5.4e1233

		Design with pre-defined morphology	Design of magnetic profile and morphology
	Magnetic profile discretization	6 discrete primary cartesian axis directions	
Demonstrations	Fig. S15	1.1e35	2.9e66
	Fig. 4	4.4e190	7.8e361
	Fig. S17	1.7e490	3.9e930
	Fig. S18	2.8e233	9.4e486

Table S3. Design space comparisons of the demonstrations. The design spaces with and without the consideration of the morphology (structural design) for magnetization directions discretized in 1 degree and 5 degrees for single magnetic material demonstrations, and in 6 discrete primary cartesian axis directions for the multi-material demonstrations, highlighting the expansion of the design space with combined magnetic programming and structural design of the robots.

<table>
<tr><th rowspan="2">Material type</th><th colspan="4">Material property</th></tr>
<tr><th>E (kPa)</th><th>Density (kg/m^3)</th><th>M_r (kA/m)</th><th>Magnetic particle mass ratio</th></tr>
<tr><td>Ecoflex 00-30</td><td>84</td><td>1.07e3</td><td>0.0</td><td>N.A.</td></tr>
<tr><td>Smooth-Sil 960</td><td>1.93e3</td><td>1.45e3</td><td>0.0</td><td>N.A.</td></tr>
<tr><td>$Nd_2Fe_{14}B$ MQFP 10-8 Ecoflex 00-30</td><td rowspan="2">200 (exp. meas.)
150 (sim. fitted*)</td><td rowspan="2">2.41e3</td><td>83 (exp. meas.)
58 (sim. fitted*)</td><td rowspan="2">2:1</td></tr>
<tr><td>$Nd_2Fe_{14}B$ MQFP 10-8 Ecoflex 00-30 (Magnetized via heat-assisted method**)</td><td>29 (exp. meas.)
20 (sim. fitted*)</td></tr>
<tr><td>$Nd_2Fe_{14}B$ MQFP 15-7 Dragonskin 30***</td><td>710</td><td>1.86e3</td><td>62</td><td>1:1</td></tr>
<tr><td>Thermo-responsive****</td><td>R.T.: 1e3
Heated: 50</td><td>1.2e3</td><td>0</td><td>N.A.</td></tr>
<tr><td>Magneto-thermo-responsive****</td><td>R.T.: 2e3
Heated: 100</td><td>2e3</td><td>60</td><td>1:1</td></tr>
</table>

Table S4. Mechanical and magnetic properties of the utilized materials. The mechanical and magnetic properties of Elastic modulus (*E*), density, magnetization strength (M), and magnetic particles to polymer mass ratio for Ecoflex 00-30, Smooth-Sil 960, mix of $Nd_2Fe_{14}B$ MQFP 10-8 & Ecoflex 00-30, mix of $Nd_2Fe_{14}B$ MQFP 15-7 & Dragonskin 30, Thermo-responsive and Magneto-thermo-responsive materials. N.A.: Not applicable, R.T.: Room temperature, *Fitted material parameters during the simulation validation for improved accuracy (Fig. S20, and SI S3), **Magnetic material fabricated via heat-assisted magnetic programming (Fig. S2), ***Taken from literature[12], ****Material properties utilized for the multifunctional magneto- and thermo-responsive soft millirobot simulations, adapted from[83].

<table>
<tr><th>Demo</th><th># of Mat.</th><th>Str. Dim.</th><th>Voxel size</th><th>Workspace</th><th>Voxel #</th><th>Seg. #</th><th>Param. #</th></tr>
<tr><td>Fig. 1F-G</td><td rowspan="5">1</td><td rowspan="6">2D</td><td rowspan="5">200 µm</td><td>6 mm x
1 mm x
200 µm</td><td>30 x 5 x 1</td><td>10 x 1 x 1</td><td>160</td></tr>
<tr><td>Fig. 2A-C
Fig. S9A-B</td><td rowspan="2">12 mm x
1 mm x
200 µm</td><td rowspan="2">60 x 5 x 1</td><td rowspan="2">20 x 1 x 1</td><td>320</td></tr>
<tr><td>Fig. 2D-E
Fig. S9C-D
Fig. S12A</td><td>340</td></tr>
<tr><td>Fig. 3A
Fig. S13B</td><td>6 mm x
6 mm x
200 µm</td><td>30 x 30 x 1</td><td>10 x 10 x 1</td><td>1100</td></tr>
<tr><td>Fig. 3D</td><td>3.6 mm x
1.4 mm x
200 µm</td><td>18 x 7 x 1</td><td>6 x 1 x 1</td><td>132</td></tr>
<tr><td>Fig. S12B</td><td rowspan="4">4</td><td rowspan="5">2 mm</td><td>10 mm x
10 mm x
200 µm</td><td>50 x 50 x 1</td><td>10 x 10 x 1</td><td>2700</td></tr>
<tr><td>Fig. S15</td><td rowspan="4">3D</td><td>18 mm x
10 mm x
2 mm</td><td>9 x 5 x 1</td><td>9 x 5 x 1</td><td>90</td></tr>
<tr><td>Fig. 4</td><td>14 mm x
14 mm x
10 mm</td><td>7 x 7 x 5</td><td>7 x 7 x 5</td><td>490</td></tr>
<tr><td>Fig. S17</td><td>14 mm x
6 mm x
60 mm</td><td>7 x 3 x 30</td><td>7 x 3 x 30</td><td>1260</td></tr>
<tr><td>Fig. S18</td><td>6</td><td>20 mm x
20 mm x
6 mm</td><td>10 x 10 x 3</td><td>10 x 10 x 3</td><td>600</td></tr>
</table>

Table S5. Details of the various parameters for the demonstrations. Parameters of number of materials employed in design (# of Mat.), structural dimensionality (Str. Dim.), voxel size, workspace dimensions available for design, voxel numbers (Voxel #), magnetic segment numbers (Seg. #), and total parameter numbers (Param. #) for the demonstrations.

Demos	Total iter. #	Total eval. #	Total time (p.i.) (sec)	Design alteration time (p.i.) (sec)	Eval. time (p.i.) (sec)	Obj. calc. (p.i.) (sec)	Archive operation (p.i.) (sec)
**Fig. 1G**	5e3	2.5e5	39±11	6±5	22±8.3	5.2±1.6	3±4.2
**Fig. 2A**	5e3	2.5e5	164±51	19±20	68±12	12.9±5.5	5.7±1.8
**Fig. 2B**	1e4	5e5	127±31	21±14	75±10	17±5.9	6.7±4.6
**Fig. 2C**	5e3	2.5e5	132±39	7.7±7.1	113±36	5.7±2.6	2.7±3.1
**Fig. 2D**	5e3	2.5e5	108±56	14±13	69±26	11±5.6	6.5±15
**Fig. 2E**	5e3	2.5e5	193±79	7.9±7.5	170±68	5.8±2.5	3.1±6.3
**Fig. 3A**	5e3	2.5e5	365±164	24±25	315±157	17±7.5	2.9±3
**Fig. 3D**	5e3	2.5e5	39±9	5.9±4.9	22±5.3	4.7±1.7	3.5±5.4
**Fig. 4C**	5e3	2.5e5	44±28	7±5	22±6	7.8±2.8	1.9±2.5
**Fig. 4D**	5e3	2.5e5	54±31	7.5±5.8	31±9	7.6±3	1.9±2.6
**Fig. 4E**	1e4	5e5	113±43	9.7±5.9	81±11	11±4.4	2.9±1.2
**Fig. S9A**	2e3	1e5	166±54	5.3±3.8	144±51	6.2±2.1	6.5±7.8
**Fig. S9B**	5e3	2.5e5	115±30	15±14	78±15	12±5.3	5.1±5.6
**Fig. S9C**	1.5e4	7.5e5	166±69	31±23	88±18	25±10	8.2±8.7
**Fig. S9D**	5e3	2.5e5	133±43	8.1±7.6	110±31	6.1±2.9	3.4±7.1
**Fig. S12A**	5e3	2.5e5	150±44	18±15	102±16	15±6.1	6.4±13
**Fig. S12B**	4e3	2e5	739±303	17±11	581±207	32±20.5	4.6±7
**Fig. S13B**	2.5e3	1.25e5	336±168	8.8±12	315±166	5.8±2.5	2.2±4.3
**Fig. S15**	5e3	2.5e5	30±8.8	4.6±3.5	17±5.2	4.2±1.1	2.5±2.5
**Fig. S17**	5e3	2.5e5	58±75	9.7±6.7	24±7.9	11±4.1	1.6±1.7
**Fig. S18**	5e3	2.5e5	282±82	7.5±6.9	255±72	9.3±4.5	1.9±3.2

Table S6. Details on the total iteration, and evaluation number, as well as, algorithm run times for all the demonstrations. The total iteration number (total iter. #), and total evaluation number (total eval. #) (50 times of the total iteration number), as well as, total wall-clock time of the algorithm, and its selected sub-modules per iteration (p.i.), including the total time per iteration (p.i.), design alteration time per iteration, evaluation time in simulation per iteration (eval. time p.i.), objective function calculation time per iteration (Obj. calc. time p.i.), and archive operation time per iteration are shown. The mean and standard deviation (std) values

are provided in (mean±std) format, and calculated by involving all the recorded times for iterations.

Supplementary references

1 Xia, X., Spadaccini, C. M. & Greer, J. R. Responsive materials architected in space and time. *Nature Reviews Materials* **7**, 683-701 (2022). https://doi.org/10.1038/s41578-022-00450-z

2 Stanley, K. O. Compositional pattern producing networks: A novel abstraction of development. *Genetic Programming and Evolvable Machines* **8**, 131-162 (2007). https://doi.org/10.1007/s10710-007-9028-8

3 Gauci, J. & Stanley, K. O. Autonomous evolution of topographic regularities in artificial neural networks. *Neural Comput.* **22**, 1860–1898 (2010). https://doi.org/10.1162/neco.2010.06-09-1042

4 Auerbach, J. E. & Bongard, J. C. Evolving CPPNs to grow three-dimensional physical structures. *Proceedings of the 12th annual conference on Genetic and evolutionary computation*, 627–634 (2010). https://doi.org/10.1145/1830483.1830597

5 Kriegman, S., Blackiston, D., Levin, M. & Bongard, J. A scalable pipeline for designing reconfigurable organisms. *Proceedings of the National Academy of Sciences* **117**, 1853-1859 (2020). https://doi.org/10.1073/pnas.1910837117

6 Cheney, N., MacCurdy, R., Clune, J. & Lipson, H. Unshackling evolution: evolving soft robots with multiple materials and a powerful generative encoding. *SIGEVOlution* **7**, 11–23 (2014). https://doi.org/10.1145/2661735.2661737

7 Cheney, N., Bongard, J. & Lipson, H. Evolving Soft Robots in Tight Spaces. *Proceedings of the 2015 Annual Conference on Genetic and Evolutionary Computation*, 935–942 (2015). https://doi.org/10.1145/2739480.2754662

8 Alapan, Y., Karacakol, A. C., Guzelhan, S. N., Isik, I. & Sitti, M. Reprogrammable shape morphing of magnetic soft machines. *Science Advances* **6**, eabc6414 (2020). https://doi.org/doi:10.1126/sciadv.abc6414

9 Cui, J. *et al.* Nanomagnetic encoding of shape-morphing micromachines. *Nature* **575**, 164-168 (2019). https://doi.org/10.1038/s41586-019-1713-2

10 Kim, Y., Yuk, H., Zhao, R., Chester, S. A. & Zhao, X. Printing ferromagnetic domains for untethered fast-transforming soft materials. *Nature* **558**, 274-279 (2018). https://doi.org/10.1038/s41586-018-0185-0

11 Xu, T., Zhang, J., Salehizadeh, M., Onaizah, O. & Diller, E. Millimeter-scale flexible robots with programmable three-dimensional magnetization and motions. *Science Robotics* **4**, eaav4494 (2019). https://doi.org/doi:10.1126/scirobotics.aav4494

12 Zhang, J. *et al.* Voxelated three-dimensional miniature magnetic soft machines via multimaterial heterogeneous assembly. *Science Robotics* **6**, eabf0112 (2021). https://doi.org/doi:10.1126/scirobotics.abf0112

13 Kim, Y. & Zhao, X. Magnetic Soft Materials and Robots. *Chemical Reviews* **122**, 5317-5364 (2022). https://doi.org/10.1021/acs.chemrev.1c00481

14 Bengio, Y., Courville, A. & Vincent, P. Representation Learning: A Review and New Perspectives. *IEEE Trans. Pattern Anal. Mach. Intell.* **35**, 1798–1828 (2013). https://doi.org/10.1109/tpami.2013.50

15 Hinton, G. E. & Salakhutdinov, R. R. Reducing the Dimensionality of Data with Neural Networks. *Science* **313**, 504-507 (2006). https://doi.org/doi:10.1126/science.1127647

16 Kingma, D. P. & Welling, M. Auto-Encoding Variational Bayes. *International Conference on Learning Representations* (2014). https://doi.org/10.48550/arXiv.1312.6114

17 Chandrasekhar, A. & Suresh, K. TOuNN: Topology Optimization using Neural Networks. *Structural and Multidisciplinary Optimization* **63**, 1135-1149 (2021). https://doi.org/10.1007/s00158-020-02748-4

18 Li, Y., Coros, S. & Thomaszewski, B. Neural Metamaterial Networks for Nonlinear Material Design. *ACM Trans. Graph.* **42**, Article 186 (2023). https://doi.org/10.1145/3618325

19 Chatzilygeroudis, K., Cully, A., Vassiliades, V. & Mouret, J.-B. Quality-Diversity Optimization: A Novel Branch of Stochastic Optimization. *Black Box Optimization, Machine Learning, and No-Free Lunch Theorems*, 109-135 (2021). https://doi.org/10.1007/978-3-030-66515-9_4

20 Mouret, J.-B. & Clune, J. Illuminating search spaces by mapping elites. *arXiv preprint arXiv:1504.04909* (2015). https://doi.org/10.48550/arXiv.1504.04909

21 Gaier, A., Asteroth, A. & Mouret, J.-B. Data-efficient design exploration through surrogate-assisted illumination. *Evol. Comput.* **26**, 381–410 (2018). https://doi.org/10.1162/evco_a_00231

22 Hagg, A., Wilde, D., Asteroth, A. & Bäck, T. Designing Air Flow with Surrogate-Assisted Phenotypic Niching. *Parallel Problem Solving from Nature – PPSN 2020, Leiden, Netherlands, September 5-9, 2020, Proceedings, Part I*, 140–153 (2020). https://doi.org/10.1007/978-3-030-58112-1_10

23 Kent, P., Gaier, A., Mouret, J.-B. & Branke, J. BOP-Elites, a Bayesian Optimisation Approach to Quality Diversity Search with Black-Box descriptor functions. *arXiv* (2023). https://doi.org/10.48550/arXiv.2307.09326

24 Lim, B., Grillotti, L., Bernasconi, L. & Cully, A. Dynamics-Aware Quality-Diversity for Efficient Learning of Skill Repertoires. *2022 International Conference on Robotics and Automation (ICRA)*, 5360–5366 (2022). https://doi.org/10.1109/icra46639.2022.9811559

25 Zhang, Y., Fontaine, M. C., Hoover, A. K. & Nikolaidis, S. Deep surrogate assisted MAP-elites for automated hearthstone deckbuilding. *Proceedings of the Genetic and Evolutionary Computation Conference*, 158–167 (2022). https://doi.org/10.1145/3512290.3528718

26 Gaier, A., Asteroth, A. & Mouret, J.-B. Discovering representations for black-box optimization. *Proceedings of the 2020 Genetic and Evolutionary Computation Conference*, 103–111 (2020). https://doi.org/10.1145/3377930.3390221

27 Rakicevic, N., Cully, A. & Kormushev, P. Policy manifold search: exploring the manifold hypothesis for diversity-based neuroevolution. *Proceedings of the Genetic and Evolutionary Computation Conference*, 901–909 (2021). https://doi.org/10.1145/3449639.3459320

28 Colas, C., Madhavan, V., Huizinga, J. & Clune, J. Scaling MAP-Elites to deep neuroevolution. *Proceedings of the 2020 Genetic and Evolutionary Computation Conference*, 67–75 (2020). https://doi.org/10.1145/3377930.3390217

29 Fontaine, M. C., Togelius, J., Nikolaidis, S. & Hoover, A. K. Covariance matrix adaptation for the rapid illumination of behavior space. *Proceedings of the 2020*

Genetic and Evolutionary Computation Conference, 94–102 (2020). https://doi.org/10.1145/3377930.3390232

30 Cully, A. Multi-emitter MAP-elites: improving quality, diversity and data efficiency with heterogeneous sets of emitters. *Proceedings of the Genetic and Evolutionary Computation Conference*, 84–92 (2021). https://doi.org/10.1145/3449639.3459326

31 Fontaine, M. & Nikolaidis, S. Differentiable quality diversity. *Advances in Neural Information Processing Systems* **34**, 10040-10052 (2021). https://doi.org/10.48550/arXiv.2106.03894

32 Nilsson, O. & Cully, A. Policy gradient assisted MAP-Elites. *Proceedings of the Genetic and Evolutionary Computation Conference*, 866–875 (2021). https://doi.org/10.1145/3449639.3459304

33 Hansen, N. The CMA Evolution Strategy: A Tutorial. *arXiv preprint arXiv:1604.00772* (2016). https://doi.org/10.48550/arXiv.1604.00772

34 Cully, A., Clune, J., Tarapore, D. & Mouret, J.-B. Robots that can adapt like animals. *Nature* **521**, 503-507 (2015). https://doi.org/10.1038/nature14422

35 Cazenille, L., Bredeche, N. & Aubert-Kato, N. Exploring Self-Assembling Behaviors in a Swarm of Bio-micro-robots using Surrogate-Assisted MAP-Elites. *2019 IEEE Symposium Series on Computational Intelligence (SSCI)*, 238-246 (2019). https://doi.org/10.1109/SSCI44817.2019.9003047

36 Fontaine, M. & Nikolaidis, S. A quality diversity approach to automatically generating human-robot interaction scenarios in shared autonomy. *Robotics: Science and Systems (RSS)* (2021). https://doi.org/10.48550/arXiv.2012.04283

37 Gravina, D., Khalifa, A., Liapis, A., Togelius, J. & Yannakakis, G. N. Procedural Content Generation through Quality Diversity. *2019 IEEE Conference on Games (CoG)*, 1–8 (2019). https://doi.org/10.1109/cig.2019.8848053

38 Fontaine, M. C. *et al.* Illuminating mario scenes in the latent space of a generative adversarial network. *Proceedings of the AAAI Conference on Artificial Intelligence* **35**, 5922-5930 (2021).

39 Jiang, Y. *et al.* An artificial intelligence enabled chemical synthesis robot for exploration and optimization of nanomaterials. *Science Advances* **8**, eabo2626 (2022). https://doi.org/doi:10.1126/sciadv.abo2626

40 Verhellen, J. & Van den Abeele, J. Illuminating elite patches of chemical space. *Chemical Science* **11**, 11485-11491 (2020). https://doi.org/10.1039/D0SC03544K

41 Galanos, T., Liapis, A., Yannakakis, G. N. & Koenig, R. ARCH-Elites: quality-diversity for urban design. *Proceedings of the Genetic and Evolutionary Computation Conference Companion*, 313–314 (2021). https://doi.org/10.1145/3449726.3459490

42 Keller, L., Tanneberg, D., Stark, S. & Peters, J. Model-based quality-diversity search for efficient robot learning. *2020 IEEE/RSJ International Conference on Intelligent Robots and Systems (IROS)*, 9675-9680 (2020).

43 Seungsu, K., Alexandre, C. & Stephane, D. From exploration to control: Learning object manipulation skills through novelty search and local adaptation. *Robotics and Autonomous Systems* **136**, 103710 (2021). https://doi.org/10.1016/j.robot.2020.103710

44 Sun, X. *et al.* Machine Learning-Evolutionary Algorithm Enabled Design for 4D-Printed Active Composite Structures. *Advanced Functional Materials* **32**, 2109805 (2022). https://doi.org/10.1002/adfm.202109805

45 Sun, X., Zhou, K., Demoly, F., Zhao, R. R. & Qi, H. J. Perspective: Machine Learning in Design for 3D/4D Printing. *Journal of Applied Mechanics* **91** (2023). https://doi.org/10.1115/1.4063684

46 Ma, C., Chang, Y., Wu, S. & Zhao, R. R. Deep Learning-Accelerated Designs of Tunable Magneto-Mechanical Metamaterials. *ACS Applied Materials \& Interfaces* **14**, 33892-33902 (2022). https://doi.org/10.1021/acsami.2c09052

47 Wu, S. *et al.* Evolutionary Algorithm-Guided Voxel-Encoding Printing of Functional Hard-Magnetic Soft Active Materials. *Advanced Intelligent Systems* **2**, 2000060 (2020). https://doi.org/10.1002/aisy.202000060

48 Chen, C.-T. & Gu, G. X. Generative Deep Neural Networks for Inverse Materials Design Using Backpropagation and Active Learning. *Advanced Science* **7**, 1902607 (2020). https://doi.org/10.1002/advs.201902607

49 Forte, A. E. *et al.* Inverse Design of Inflatable Soft Membranes Through Machine Learning. *Advanced Functional Materials* **32**, 2111610 (2022). https://doi.org/10.1002/adfm.202111610

50 Pugh, J. K., Soros, L. B., Szerlip, P. A. & Stanley, K. O. Confronting the Challenge of Quality Diversity. *Proceedings of the 2015 Annual Conference on Genetic and Evolutionary Computation*, 967–974 (2015). https://doi.org/10.1145/2739480.2754664

51 Hiller, J. & Lipson, H. Dynamic Simulation of Soft Multimaterial 3D-Printed Objects. *Soft Robotics* **1**, 88-101 (2014). https://doi.org/10.1089/soro.2013.0010

52 The GPyOpt authors. *GPyOpt : A Bayesian Optimization framework in python*, <http://github.com/SheffieldML/GPyOpt> (2016).

53 Hu, W., Lum, G. Z., Mastrangeli, M. & Sitti, M. Small-scale soft-bodied robot with multimodal locomotion. *Nature* **554**, 81-85 (2018). https://doi.org/10.1038/nature25443

54 Savitzky, A. & Golay, M. J. E. Smoothing and Differentiation of Data by Simplified Least Squares Procedures. *Analytical Chemistry* **36**, 1627-1639 (1964). https://doi.org/10.1021/ac60214a047

55 Lum, G. Z. *et al.* Shape-programmable magnetic soft matter. *Proceedings of the National Academy of Sciences* **113**, E6007-E6015 (2016). https://doi.org/doi:10.1073/pnas.1608193113

56 Wang, L. *et al.* Evolutionary design of magnetic soft continuum robots. *Proceedings of the National Academy of Sciences* **118**, e2021922118 (2021). https://doi.org/doi:10.1073/pnas.2021922118

57 Lloyd, P. *et al.* A Magnetically-Actuated Coiling Soft Robot With Variable Stiffness. *IEEE Robotics and Automation Letters* **8**, 3262-3269 (2023). https://doi.org/10.1109/LRA.2023.3264770

58 Tian, J., Zhao, X., Gu, X. D. & Chen, S. Designing Ferromagnetic Soft Robots (FerroSoRo) with Level-Set-Based Multiphysics Topology Optimization. *2020 IEEE International Conference on Robotics and Automation (ICRA)*, 10067-10074 (2020). https://doi.org/10.1109/ICRA40945.2020.9197457

59 Lloyd, P. *et al.* A Learnt Approach for the Design of Magnetically Actuated Shape Forming Soft Tentacle Robots. *IEEE Robotics and Automation Letters* **5**, 3937-3944 (2020). https://doi.org/10.1109/LRA.2020.2983704

60 Zhi, Z. & Xiaojia Shelly, Z. Topology optimization of hard-magnetic soft materials. *Journal of the Mechanics and Physics of Solids* **158**, 104628 (2022). https://doi.org/10.1016/j.jmps.2021.104628

61 Bacchetti, A. *et al.* Optimization and fabrication of programmable domains for soft magnetic robots: A review. *Frontiers in Robotics and AI* **9**, 1040984 (2022).

62 Ruike, Z., Yoonho, K., Shawn, A. C., Pradeep, S. & Xuanhe, Z. Mechanics of hard-magnetic soft materials. *Journal of the Mechanics and Physics of Solids* **124**, 244-263 (2019). https://doi.org/10.1016/j.jmps.2018.10.008

63 Park, S.-J. *et al.* Phototactic guidance of a tissue-engineered soft-robotic ray. *Science* **353**, 158-162 (2016). https://doi.org/doi:10.1126/science.aaf4292

64 Pikul, J. H. *et al.* Stretchable surfaces with programmable 3D texture morphing for synthetic camouflaging skins. *Science* **358**, 210-214 (2017). https://doi.org/doi:10.1126/science.aan5627

65 Boley, J. W. *et al.* Shape-shifting structured lattices via multimaterial 4D printing. *Proceedings of the National Academy of Sciences* **116**, 20856-20862 (2019). https://doi.org/doi:10.1073/pnas.1908806116

66 Guo, Y., Shahsavan, H. & Sitti, M. 3D Microstructures of Liquid Crystal Networks with Programmed Voxelated Director Fields. *Advanced Materials* **32**, 2002753 (2020). https://doi.org/10.1002/adma.202002753

67 Murphy, S. V. & Atala, A. 3D bioprinting of tissues and organs. *Nature Biotechnology* **32**, 773-785 (2014). https://doi.org/10.1038/nbt.2958

68 Athinarayanarao, D. *et al.* Computational design for 4D printing of topology optimized multi-material active composites. *npj Computational Materials* **9**, 1 (2023). https://doi.org/10.1038/s41524-022-00962-w

69 Goodfellow, I. *et al.* Generative adversarial nets. *Advances in neural information processing systems* **27** (2014).

70 Mao, Y., He, Q. & Zhao, X. Designing complex architectured materials with generative adversarial networks. *Science Advances* **6**, eaaz4169 (2020). https://doi.org/doi:10.1126/sciadv.aaz4169

71 Bonfanti, S., Guerra, R., Font-Clos, F., Rayneau-Kirkhope, D. & Zapperi, S. Automatic design of mechanical metamaterial actuators. *Nature Communications* **11**, 4162 (2020). https://doi.org/10.1038/s41467-020-17947-2

72 Wang, C., Zhao, Z., Zhou, M., Sigmund, O. & Zhang, X. S. A comprehensive review of educational articles on structural and multidisciplinary optimization. *Struct. Multidiscip. Optim.* **64**, 2827–2880 (2021). https://doi.org/10.1007/s00158-021-03050-7

73 Ruiz, D. & Sigmund, O. Optimal design of robust piezoelectric microgrippers undergoing large displacements. *Structural and Multidisciplinary Optimization* **57**, 71-82 (2018). https://doi.org/10.1007/s00158-017-1863-5

74 Geiss, M. J., Boddeti, N., Weeger, O., Maute, K. & Dunn, M. L. Combined Level-Set-XFEM-Density Topology Optimization of Four-Dimensional Printed Structures Undergoing Large Deformation. *Journal of Mechanical Design* **141** (2019). https://doi.org/10.1115/1.4041945

75 Lumpe, T. S. & Shea, K. Computational design of 3D-printed active lattice structures for reversible shape morphing. *Journal of Materials Research* **36**, 3642-3655 (2021). https://doi.org/10.1557/s43578-021-00225-2

76 Sundaram, S., Skouras, M., Kim, D. S., van den Heuvel, L. & Matusik, W. Topology optimization and 3D printing of multimaterial magnetic actuators and displays. *Science Advances* **5**, eaaw1160 (2019). https://doi.org/doi:10.1126/sciadv.aaw1160

77 Changyoung, Y., Yuki, S., Hiroki, K., Atsushi, K. & Tsuyoshi, N. 4D topology optimization: Integrated optimization of the structure and self-actuation of soft bodies for dynamic motions. *Computer Methods in Applied Mechanics and Engineering* **414**, 116187 (2023). https://doi.org/10.1016/j.cma.2023.116187

78 Shin, S., Shin, D. & Kang, N. Topology optimization via machine learning and deep learning: A review. *Journal of Computational Design and Engineering* **10**, 1736-1766 (2023).

79 Woldseth, R. V., Aage, N., Bærentzen, J. A. & Sigmund, O. On the use of artificial neural networks in topology optimisation. *Structural and Multidisciplinary Optimization* **65**, 294 (2022). https://doi.org/10.1007/s00158-022-03347-1

80 Frazier, P. I. A tutorial on Bayesian optimization. *arXiv preprint arXiv:1807.02811* (2018). https://doi.org/10.48550/arXiv.1807.02811

81 Snoek, J., Larochelle, H. & Adams, R. P. Practical bayesian optimization of machine learning algorithms. *Advances in neural information processing systems* **25** (2012).

82 *miniball*, <https://pypi.org/project/miniball> (2024).

83 Ze, Q. *et al.* Magnetic Shape Memory Polymers with Integrated Multifunctional Shape Manipulation. *Advanced Materials* **32**, 1906657 (2020). https://doi.org/10.1002/adma.201906657
