# Supplementary material for: Data-driven design of shape-programmable magnetic soft materials
Source: Nat Commun. 2025 Mar 26;16:2946. doi: 10.1038/s41467-025-58091-z (PMC11947188; doi:10.1038/s41467-025-58091-z)
Supplement: Supplementary file 2 — Description of Additional Supplementary Files [file 41467_2025_58091_MOESM2_ESM.pdf]

Description of Additional Supplementary Files

Supplementary Movie 1 - 2D shape-morphing: Data driven design of morphology and magnetic profile of magnetic soft beams for 2D shapes.

Supplementary Movie 2 - 3D shape-morphing: Data driven design of morphology and magnetic profile of magnetic soft beams for 3D shapes.

Supplementary Movie 3 - Morphological behavior tasks: Data-driven design of morphological tasks in magnetic soft structures.

Supplementary Movie 4 - Jumping behavior: Data-driven design of jumping behavior in magnetic soft millirobots.

Supplementary Movie 5 - Multi-material and 3D structural design: Data-driven design of magnetic soft millirobots with multi-material and inherently 3D structure.

Supplementary Movie 6 – Configurable behavior: Design of configurable magneto- and thermo-responsive soft millirobots with 3D multi-material composition.
